# Cofilin1 Controls Transcolumnar Plasticity in Dendritic Spines in Adult Barrel Cortex

**DOI:** 10.1371/journal.pbio.1002070

**Published:** 2015-02-27

**Authors:** Tadashi Tsubota, Reiko Okubo-Suzuki, Yohei Ohashi, Keita Tamura, Koshin Ogata, Masae Yaguchi, Makoto Matsuyama, Kaoru Inokuchi, Yasushi Miyashita

**Affiliations:** 1 Department of Physiology, The University of Tokyo School of Medicine, Tokyo, Japan; 2 Department of Biochemistry, Graduate School of Medicine and Pharmaceutical Sciences, University of Toyama, Toyama, Japan; 3 Core Research for Evolutional Science and Technology (CREST), Japan Science and Technology Agency (JST), Saitama, Japan; Max-Planck Institute of Neurobiology, GERMANY

## Abstract

During sensory deprivation, the barrel cortex undergoes expansion of a functional column representing spared inputs (spared column), into the neighboring deprived columns (representing deprived inputs) which are in turn shrunk. As a result, the neurons in a deprived column simultaneously increase and decrease their responses to spared and deprived inputs, respectively. Previous studies revealed that dendritic spines are remodeled during this barrel map plasticity. Because cofilin1, a predominant regulator of actin filament turnover, governs both the expansion and shrinkage of the dendritic spine structure *in vitro*, it hypothetically regulates both responses in barrel map plasticity. However, this hypothesis remains untested. Using lentiviral vectors, we knocked down cofilin1 locally within layer 2/3 neurons in a deprived column. Cofilin1-knocked-down neurons were optogenetically labeled using channelrhodopsin-2, and electrophysiological recordings were targeted to these knocked-down neurons. We showed that cofilin1 knockdown impaired response increases to spared inputs but preserved response decreases to deprived inputs, indicating that cofilin1 dependency is dissociated in these two types of barrel map plasticity. To explore the structural basis of this dissociation, we then analyzed spine densities on deprived column dendritic branches, which were supposed to receive dense horizontal transcolumnar projections from the spared column. We found that spine number increased in a cofilin1-dependent manner selectively in the distal part of the supragranular layer, where most of the transcolumnar projections existed. Our findings suggest that cofilin1-mediated actin dynamics regulate functional map plasticity in an input-specific manner through the dendritic spine remodeling that occurs in the horizontal transcolumnar circuits. These new mechanistic insights into transcolumnar plasticity in adult rats may have a general significance for understanding reorganization of neocortical circuits that have more sophisticated columnar organization than the rodent neocortex, such as the primate neocortex.

## Abstract 2

In vivo measurement of the electrophysiology and shape of neurons reveals that cofilin1 is needed for remodeling dendritic spines in circuits that connect mouse whisker barrels, so aiding experience-dependent plasticity in the neocortex.

## Author Summary

Plasticity in the adult neocortex is the basis of our learning and memory. However, its molecular mechanisms are still unclear. In the sensory barrel cortex of rodents, a well-characterized model for neocortical plasticity, neurons directly code for whisker displacement—neurons within a given barrel will fire when the whisker that that barrel represents is moved. Strikingly, the deprivation of all but a single whisker alters the original representations—cortical columns representing the deprived inputs shrink and that representing the spared inputs expands, intruding into the surrounding deprived columns. Because single-neuron-level structural changes are suggested to be involved in this plasticity, here we focused on cofilin1, a protein that is known to modulate the cytoskeleton and to regulate the structure of dendritic spines. We induced experience-dependent plasticity in the D1 column by sparing only the D1 whisker, and knocked down the expression of cofilin1 in the D2 column. Cofilin1 knockdown differentially affected plasticity, such that experience-dependent increases in spared input representation were impaired, whereas decreases in deprived input representation were intact. We then found that during these plastic changes, the density of dendritic spines increased in a cofilin1-dependent manner around the connections between the D1 and D2 columns. Cofilin1-dependent density increase was observed only in the most superficial part of the cortex but not in deeper parts, consistent with the distribution patterns of axons that transmit spared and deprived information, respectively. These results suggest that cofilin1 regulates neocortical functional plasticity through the remodeling of dendritic spines within circuits that connect columns.

## Introduction

Experience-dependent plasticity (EDP) in adult neuronal circuits is considered to form the basis of learning and memory [1–3]. EDP is also related to the recovery of cortical responses after the disruption of peripheral inputs [4–6]. The rodent barrel cortex provides a key model system for studying adult EDP in which response field patterns (or functional maps) can change in a sensory experience-dependent fashion [7–9]. In this regard, the response field corresponding to a spared whisker (spared cortical barrel column) expands during sensory deprivation into neighboring cortical columns that correspond to deprived whiskers (deprived columns) [10]. The deprived inputs associated with these columns in turn shrink [11]. The primary locus of these plastic changes is layer 2/3 (L2/3) in adult barrel cortex [12,13]. An L2/3 neuron increases its responses to spared inputs and simultaneously decreases its responses to deprived inputs [14].

These plastic changes have been suggested to be mediated by different neuronal circuits. Horizontal transcolumnar projections from neighboring column neurons (spared column → deprived column L2/3) [15,16] are important for map expansion because electrolytic lesioning of this pathway prevents plasticity [17] and synaptic transmission in this pathway is potentiated after sensory deprivation [18]. In contrast, ascending intracolumnar projections from layer 4 (deprived column L4 → deprived column L2/3) are depressed during shrinkage of deprived whisker representation [19].

Morphological studies indicate that sensory deprivation promotes the turnover of dendritic spines in the rodent barrel cortex [20]. Within dendritic spines, actin filaments are highly concentrated [21,22] and provide the structural foundation for synaptic plasticity [23–25]. Actin depolymerizing factor (ADF)/cofilin regulate dendritic spine structure through their actin filament-severing and monomer-binding activities [26]. ADF/cofilin are thus predominant regulators of dendritic spine structure and synaptic plasticity [27–32]. Indeed, ADF/cofilin govern both the expansion and shrinkage of the spine structure of the hippocampal neuronal dendrite *in vitro* [28,33], and postnatal knockout impairs both stimulus-induced long-term potentiation (LTP) and depression (LTD) in hippocampus [31]. However, it remains untested whether ADF/cofilin regulate both directions of response changes during adult EDP. In the present study, therefore, we investigated causal impact of perturbation of ADF/cofilin function on two different components of barrel map plasticity: spared input expansion and deprived input shrinkage.

**Fig 1:**
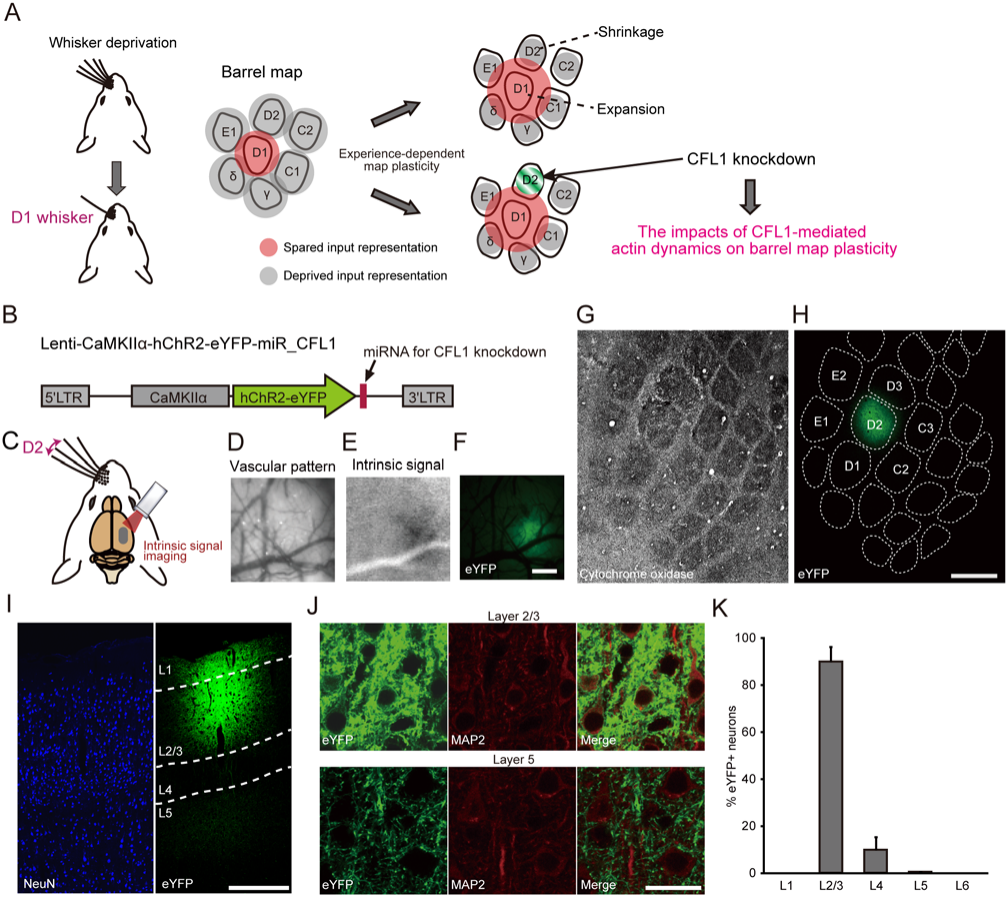
L2/3- and D2 column-restricted miRNA expression achieved through focal lentiviral injection. (A) The impact of CFL1 on barrel map plasticity was examined through focal CFL1 KD within L2/3 of the D2 column. (B) The lentiviral vectors employed in this study for co-expressing either miRNA against CFL1 (miR-CFL1_1 or miR-CFL1_2) or miR-Neg and ChR2-eYFP. LTR, long terminal repeat. (C) A schematic diagram of intrinsic signal optical imaging of the right barrel cortex during left D2 whisker stimulation. (D, E) A vascular pattern image (D) and an intrinsic signal induced by D2 stimulation (E) during a representative imaging session. (F) An eYFP fluorescence image of the same cortical area shown in (D and E). Scale bar, 500 μm. (G, H) Tangential sections corresponding to L4 (G) and L2/3 (H) of a rat that was injected lentivirus into the D2 column. The L4 section was stained for cytochrome oxidase to visualize the barrel pattern that was projected onto the L2/3 image based on the vascular pattern. Scale bar, 500 μm. (I) A NeuN-stained coronal section of a virus-injected rat. Scale bar, 300 μm. (J) Magnified images of L2/3 (top row) and L5 (bottom row) of a MAP2 stained coronal section. Scale bar, 20 μm. (K) Percentage of eYFP+ neurons in each cortical layer. *n* = 5 injections.

For this purpose, we knocked down cofilin1 (CFL1) within excitatory neuron in an L2/3- and deprived (D2) column-restricted manner (Fig. 1A). This strategy enabled us to examine the impact of CFL1 knockdown (KD) only in the direct postsynaptic neurons (in this case, the L2/3 excitatory neurons of the deprived column) involved in the horizontal transcolumnar and ascending intracolumnar connections. We found that the response field expansion of spared whisker input was impaired by CFL1 KD in the deprived column, while the response field shrinkage of deprived whisker input was preserved. We then explored the mechanistic insights for this dissociation in the effects of CFL1 KD, and found that spine densities increased in a CFL1-dependent manner at the dendritic branch segments around spines connecting with transcolumnar projections, selectively in a part of the supragranular layer where dense transcolumnar projections were observed. These results provide the first direct evidence that a CFL1-mediated change in synaptic connectivity underlies the EDP in a circuit-specific manner.

## Results

### D2 Column- and L2/3-Restricted Expression of MicroRNA Targeting *CFL1* Gene

To manipulate *CFL1* gene expression, we used a microRNA (miRNA)-based gene KD system in which the polymerase II promoter was available for driving miRNA expression. Because several weeks are typically required for the induction of EDP [10,14], we employed a lentiviral vector for the stable *in vivo* expression of miRNA for CFL1 KD (Fig. 1B). The vectors co-expressed channelrhodopsin-2 (ChR2)-enhanced yellow fluorescent protein (eYFP), with ChR2 as a light-activatable tag for extracellular single-unit recordings [34–38] and eYFP as a fluorescent marker of CFL1 KD neurons, under the control of the excitatory neuron specific Ca^2+^/calmodulin-dependent protein kinase II alpha (CaMKIIα) promoter [39].

Targeted injection of the lentiviral vector into the right D2 barrel column of rats was achieved by functionally identifying the column center via intrinsic signal optical imaging (Fig. 1C-1F) [40]. Single column-restricted expression of eYFP was confirmed in tangential sections that were processed via cytochrome oxidase staining (Fig. 1G and 1H). By targeting the vector injection to a shallow depth within the cerebral cortex (~300 μm from the pial surface), viral infection was restricted to L2/3 (Fig. 1I), and strong eYFP expression was confined to L2/3 (Fig. 1J). Owing to the existence of axonal projections from L2/3 to L5 [16], weak fluorescence derived from eYFP-positive axons originating from L2/3 neurons was observed in L5 (Fig. 1J). Immunostaining of a neuronal marker, microtubule-associated protein 2 (MAP2), confirmed that most of the eYFP+ neurons resided within L2/3, whereas eYFP+ neurons were rarely found in L5 (90.1% in L2/3 versus 0.2% in L5) (Fig. 1J and 1K). These results demonstrate that viral expression was mostly restricted to the L2/3 neurons in the D2 barrel column.

### Efficiency and Specificity of miR-CFL1

**Fig 2:**
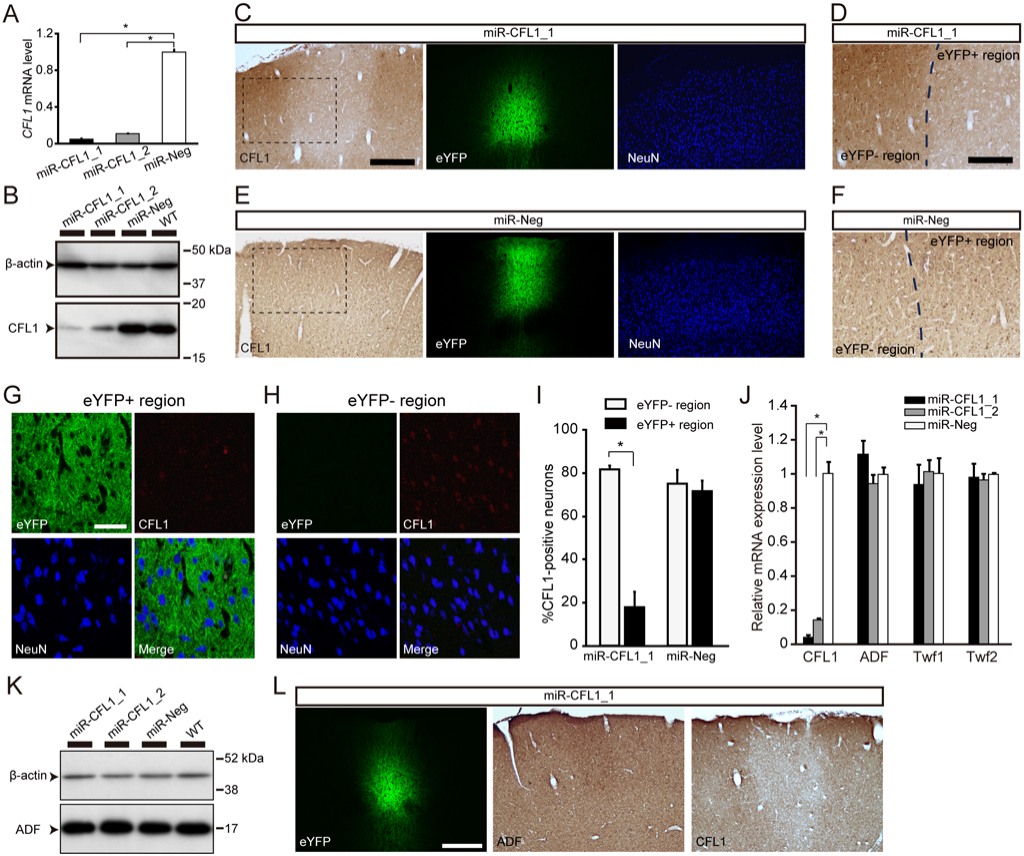
Efficiency and specificity of CFL1 KD through miR-CFL1. (A) CFL1 mRNA KD efficiency of two miRNAs (miR-CFL1_1 and miR-CFL1_2) targeted against different sequences within the CFL1 gene, assessed by using CFL1-overexpressing HEK 293T-cells. CFL1 mRNA levels were normalized to those of the miR-Neg group. *n* = 3 for all groups; miR-CFL1_1, *p* = 2.7 × 10^-8^; miR-CFL1_2, *p* = 2.8 × 10^-8^ versus miR-Neg, Dunnett’s multiple comparison test. (B) KD efficiency of miR-CFL1_1 and miR-CFL1_2 for endogenous CFL1 protein, assessed by using PC-12 cells. “WT” (wild type) indicates PC-12 cells that were not infected with lentivirus. (C) Two neighboring coronal sections obtained from a miR-CFL1_1-expressing rat are shown, one stained with an antibody against CFL1 (left) and the other stained with an antibody against NeuN (right). The eYFP fluorescence image (middle) was obtained from the NeuN-stained section. Scale bar, 300 μm. (D) Magnified view of the rectangular region indicated in (C). Scale bar, 150 μm. (E, F) Same as (C and D), but of two neighboring coronal sections derived from a miR-Neg virus-injected rat. (G, H) Confocal images of eYFP+ or eYFP− region in a coronal section obtained from a miR-CFL1_1-expressing rat stained with antibodies against NeuN (blue) and CFL1 (red). Scale bar, 50 μm. (I) Percentage of CFL1+ cells in NeuN+ cells measured in miR-CFL1_1- or miR-Neg-expressing rats. *n* = 3 for all groups. *p = 0.0003, t-test with Bonferroni’s correction. (J) Effects of miR-CFL1 on mRNA expression of genes related to CFL1, assessed in PC-12 cells. *n* = 3 for all groups. miR-CFL1_1 of CFL1, *p* = 2.6 × 10^-7^; miR-CFL1_2 of CFL1, *p* = 5.2 × 10^-7^ versus miR-Neg, Dunnett’s multiple comparison test. (K) Effects of miR-CFL1 on expression of ADF protein in PC-12 cells. (L) Three successive coronal sections obtained from a miR-CFL1_1-expressing rat are shown, one stained with an antibody against ADF (middle) and another stained with an antibody against CFL1 (right). Scale bar, 300 μm.

In the present study, the CFL1 KD experiments made use of a negative control miRNA (miR-Neg) and two miRNAs (miR-CFL1_1 and miR-CFL1_2) with different target sequences within the *CFL1* gene. The KD efficiencies of miR-CFL1_1 and miR-CFL1_2 were first assessed *in vitro*. Both miRNAs showed high KD efficiency at the mRNA level in rat CFL1-overexpressing human embryonic kidney (HEK) 293T cells (miR-CFL1_1, 95.0 ± 0.9%; miR-CFL1_2, 89.3 ± 0.3%; *n* = 3: miR-CFL1_1, *p* = 2.7 × 10^-8^; miR-CFL1_2, *p* = 2.8 × 10^-8^ versus miR-Neg, Dunnett’s multiple comparison test) (Fig. 2A). Similar results were observed at the protein level in rat pheochromocytoma-12 (PC-12) cells (miR-CFL1_1, 96.8 ± 1.5%; miR-CFL1_2, 83.8 ± 5.7%; *n* = 3: miR-CFL1_1, *p* = 0.0036; miR-CFL1_2, *p* = 0.0073 versus miR-Neg) (Figs. 2B and S1A). We next confirmed CFL1 KD *in vivo*. CFL1 protein expression is observed in neuronal somata, dendritic spines, and astrocytes in the normal cortex [21]. Immunostaining of CFL1 in a miR-CFL1_1- or miR-CFL1_2-expressing rat showed that CFL1 expression decreased locally in an L2/3 subregion corresponding to the region where eYFP expression was observed in the adjacent section (Figs. 2C, 2D, and S1B). This finding is in clear contrast with the miR-Neg-expressing control section, where no decrease in CFL1 protein expression was observed (Fig. 2E and 2F). Double staining of CFL1 and NeuN in a miR-CFL1_1-expressing rat revealed that CFL1 immunoreactivity decreased in neurons within eYFP+ region compared to eYFP− region (Fig. 2G and 2H). Indeed, percentage of CFL1-positive cells in NeuN-positive cells significantly decreased in eYFP+ region (eYFP− region, 81.6 ± 1.2%; eYFP+ region, 17.8 ± 4.4%; *n* = 3; *p* = 0.0003, *t*-test with Bonferroni’s correction) (Fig. 2I). This percentage did not decrease in miR-Neg-expressing rats (eYFP− region, 75.2 ± 3.8%; eYFP+ region, 71.7 ± 3.0%; *p* = 0.51, *t*-test) (Fig. 2I). These results demonstrate that expression of miR-CFL1 knocked down CFL1 in neurons and this effect was not due to overexpression of ChR2-eYFP or miRNA.

To ensure the specificity of miR-CFL1, we next examined mRNA expression levels of three genes related to *CFL1* (*ADF*, *Twinfilin 1* and *2*) [41] in PC-12 cells. Expression of miR-CFL1 through the infection of Lenti-CMV-ChR2-eYFP-miR-CFL1_1 or-CFL1_2 did not affect the mRNA levels of these genes (*ADF*; miR-CFL1_1, *p* = 0.11; miR-CFL1_2, *p* = 0.50: *Twinfilin1*; miR-CFL1_1, *p* = 0.66; miR-CFL1_2, *p* = 0.99: *Twinfilin2*; miR-CFL1_1, *p* = 0.86; miR-CFL1_2, *p* = 0.69: versus miR-Neg, Dunnett’s multiple comparison test), although *CFL1* expression significantly decreased (miR-CFL1_1, *p* = 2.6 × 10^-7^; miR-CFL1_2, *p* = 5.2 × 10^-7^ versus miR-Neg) (Fig. 2J). As for ADF, which is closely related to CFL1 in terms of structure and function [26], we examined its expression at the protein level both in PC-12 cells and rats expressing miR-CFL1. ADF protein expression was not significantly affected by CFL1 KD (miR-CFL1_1, *p* = 0.094; miR-CFL1_2, *p* = 0.078: versus miR-Neg, Dunnett’s multiple comparison test), although there was a slight increase (Figs. 2K, 2L, and S1C). This tendency of increase is consistent with the previous observations in CFL1 knockout mice [31]. These data clearly demonstrate the specificity of genetic manipulation mediated by miR-CFL1.

### CFL1 KD Impairs Experience-Dependent Expansion of Spared Whisker Representation

In the present study, extracellular single-unit recordings were only taken from regular-spiking neurons (S2A–S2C Fig.) [42]. For efficient recording from CFL1 KD neurons that existed only within a small cortical region (approximately, a sphere with a radius of 200–250 μm) (Fig. 1H and 1I), we searched CFL1 KD neurons that co-expressed ChR2 (Fig. 1B) with illuminating blue laser (peak wavelength: 473 nm). Furthermore, to exclude neurons that weakly expressed or did not express ChR2, we used only the data of light-responsive L2/3 neurons that showed high response reliability [37] to repetitive light (20 Hz) stimulation (S2D–S2K Fig.) (for details, see Materials and Methods).

**Fig 3:**
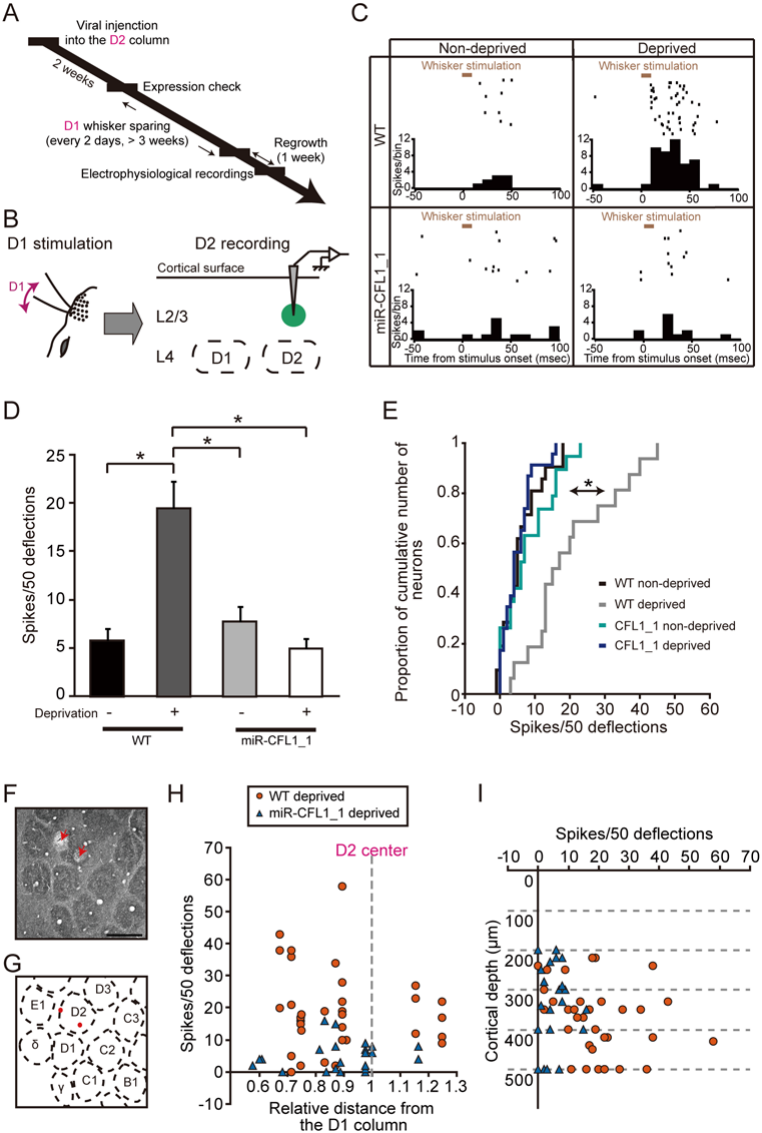
Effects of CFL1 KD on D2 neuronal responses to spared D1 inputs. (A) Experimental time course of virus injection, whisker sparing, and electrophysiological recordings. (B) Optogenetically labeled CFL1 KD neurons in L2/3 of the D2 barrel column were recorded while the D1 whisker was stimulated. (C) Example raster plots (50 trials are shown in horizontal row) and peristimulus time histograms (PSTHs) (averaged across all trials) of single neurons recorded in WT non-deprived, WT deprived, miR-CFL1_1 non-deprived, and miR-CFL1_1 deprived rat, respectively. (D) Average number of spikes measured in response to D1 whisker stimulation in D2 column L2/3 neurons for each rat group. *n* = 21, 33, 19 and 23 units for WT non-deprived, WT deprived, miR-CFL1_1 non-deprived and miR-CFL1_1 deprived groups, respectively. WT non-deprived, *p* = 2.7 × 10^-6^; miR-CFL1_1 non-deprived, *p* = 1.1 × 10^-4^; miR-CFL1_1 deprived, *p* = 3.3 × 10^-7^ versus WT deprived group, Tukey-Kramer’s multiple comparison test. (E) Cumulative frequency histogram of spike number. WT non-deprived, *p* = 2.0 × 10^-4^; miR-CFL1_1 non-deprived, *p* = 0.028; miR-CFL1_1 deprived, *p* = 1.8 × 10^-6^ versus WT deprived group, Kolmogorov-Smirnov test with Bonferroni’s correction. (F) A representative tangential section of cortical L4 in which two electrolytic lesions were made (indicated with red arrows). Scale bar, 500 μm. (G) A schematic diagram of the barrel pattern. Both lesion marks were within the D2 barrel column. (H, I) Distribution of average spike number in response to D1 whisker stimulation versus distance from the D1 column center (H) or cortical depth (I) for neurons obtained from WT and miR-CFL1_1 deprived rats.

To examine the effects of CFL1 KD on EDP, lentiviral vectors were injected at 2 weeks before the onset of sensory deprivation (Fig. 3A). Sensory deprivation was induced by using the single whisker experience protocol [7,10], in which all whiskers but the D1 whisker were trimmed on the left side of the face. We first confirmed that the L2/3 neurons in the D2 column of the right hemisphere showed increased responses to spared D1 whisker stimulation after sensory deprivation in wild-type (WT) rats (WT non-deprived versus WT deprived, *p* = 2.7 × 10^-6^, Tukey-Kramer’s multiple comparison test) (Fig. 3B-3E). This observation indicates that the cortical representation of spared whisker inputs expanded into surrounding deprived columns (Fig. 1A). In contrast, the neuronal response increase was almost completely absent in the putative ChR2+ neurons from which recordings were taken in the miR-CFL1_1-expressing deprived rats (WT deprived versus miR-CFL1_1 deprived, *p* = 3.3 × 10^-7^) (Fig. 3C-3E). On the other hand, expression of miR-CFL1_1 in non-deprived rats did not affect neuronal responses to D1 stimulation (WT non-deprived versus miR-CFL1_1 non-deprived, *p* = 0.91) (Fig. 3C-3E), indicating that CFL1 KD in and of itself does not decrease responses to the D1 whisker.

We performed three lines of control experiments. The first control experiment demonstrated that the enhanced spared whisker response was not altered in neurons in which miR-Neg and ChR2-eYFP were co-expressed (WT deprived versus miR-Neg deprived, *p* = 0.98) (S3A Fig.). This finding suggests that the overexpression of miRNA and ChR2-eYFP itself does not affect the expansion of spared input representation. The second control experiment demonstrated that CFL1 KD with miR-CFL1_2 also impaired the increase in the spared whisker response as well as miR-CFL1_1 (WT deprived versus miR-CFL1_2 deprived, *p* = 4.1 × 10^-6^) (S3A Fig.). This finding suggests that the observed effects were not due to “off-target” actions of miR-CFL1 [43]. Finally, the effect of CFL1 KD on experience-dependent response increase was weaker in neurons determined as putative ChR2− than those determined as putative ChR2+ both in miR-CFL1_1 and miR-CFL1_2 (*F*_*1*, *90*_ = 9.01, *p* = 0.0035, main effect of factor 1; factor 1, neuron type; factor 2, miR type; two-way ANOVA: miR-CFL1_1, *p* = 0.017; miR-CFL1_2, *p* = 0.15; ChR2+ versus ChR2−, *t-*test with Bonferroni’s correction) (S2L Fig.), validating further that experience-dependent potentiation to D1 deflections was impaired in miR-CFL1-expressing D2 neurons.

To exclude the possibility that biased recording locations within the D2 column affected our results, we performed another analysis. Recording locations in the D2 column were reconstructed based on lesion marks (Fig. 3F and 3G). Responses recorded from CFL1 KD neurons of deprived rats were lower than that recorded from WT deprived rats, regardless of the distance from the D1 column (Fig. 3H) and the cortical depth (Fig. 3I) (comparison of distance distribution, WT deprived versus miR-CFL1_1 deprived, *p* = 0.37; comparison of depth distribution, WT deprived versus miR-CFL1_deprived, *p* = 0.19, Mann-Whitney’s U-test). Accordingly, the observed effects of CFL1 KD in this study were not due to recording location bias.

### Impairment of Experience-Dependent Plasticity Can Be Rescued by Expression of CFL1 Resistant to miRNA

**Fig 4:**
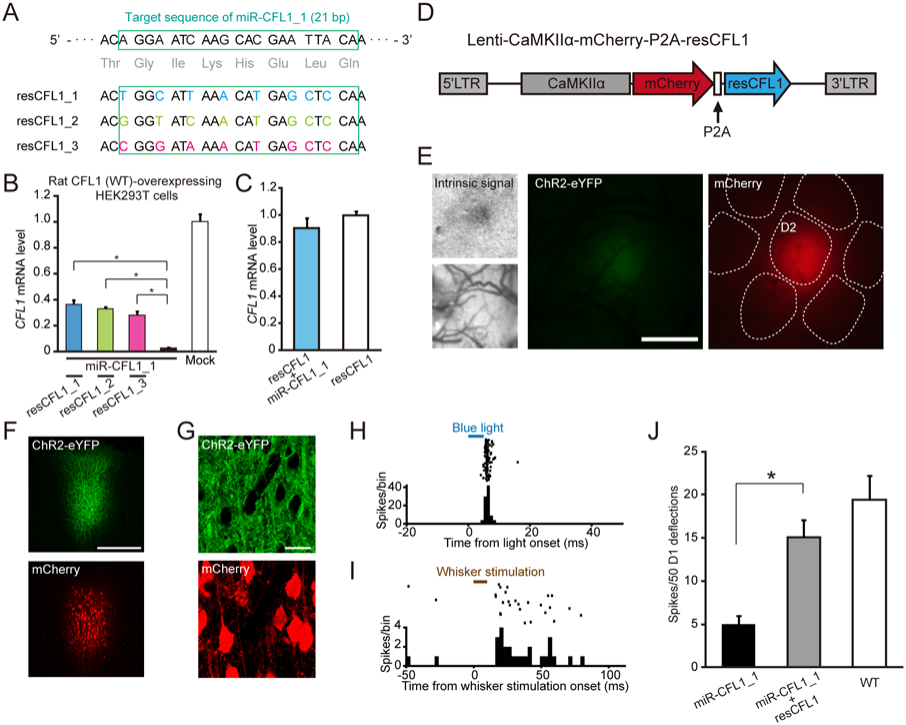
Effects of CFL1 expression rescue on impaired experience-dependent plasticity. (A) Design of three mutant CFL1s that are resistant to miR-CFL1_1 (resCFL1s). Seven or eight nucleic acids within the target sequence of miR-CFL1_1 were mutated such that amino acid sequence did not change. (B) An *in vitro* test of CFL1 expression rescue by each resCFL1 in rat CFL1 (WT)- and miR-CFL1_1-expressing HEK293T cells. The mock group expressed only WT CFL1. *n* = 4 for all groups. resCFL1_1, *p* = 5.8 × 10^-6^; resCFL1_2, *p* = 2.2 × 10^-5^; resCFL1_3, *p* = 1.3 × 10^-4^ versus miR-CFL1_1 group, Tukey-Kramer’s multiple comparison test. (C) An *in vitro* test of the effect of miR-CFL1_1 on resCFL1_1 expression. *n* = 3 for both groups. *p* = 0.30, *t*-test. (D) A schematic diagram of the bicistronic lentiviral vector that co-expresses resCFL1_1 and mCherry via a P2A peptide. (E) Targeted injection of Lenti-CaMKIIα-ChR2-eYFP-miR-CFL1_1 and Lenti-CaMKIIα-mCherry-P2A-resCFL1 to D2 column identified with intrinsic signal imaging induced focal expression of ChR2-eYFP and mCherry. Scale bar, 500 μm. (F) Fluorescent images of a coronal section infected with the two vectors. Scale bar, 300 μm. (G) Confocal images of an infected area showing co-expression of ChR2-eYFP and mCherry in L2/3 neurons. Scale bar, 20 μm. (H) A representative raster plot (100 trials are shown in horizontal row) and peristimulus time histogram (PSTH) of a putative ChR2+ neuron recorded from L2/3 in D2 column of the rat showed in E. (I) A representative raster plot (50 trials) and PSTH of the same neuron with (H), showing responses to D1 whisker deflections. (J) Comparison of average responses recorded from ChR2+ neurons in D2 L2/3 of deprived rats expressing miR-CFL1_1 and resCFL1 with those recorded from ChR2+ neurons in deprived rats expressing only miR-CFL1_1 and those recorded from WT deprived rats. Data of miR-CFL1_1 deprived and WT deprived groups were the same with those shown in Fig. 3D. *n* = 17 units (from three rats) for the miR-CFL1_1+resCFL1 deprived group. *p = 0.0069, Tukey-Kramer’s multiple comparison test.

To further confirm the specificity of the effects of miR-CFL1, we next examined whether the impaired experience-dependent increase in neuronal responses (Fig. 3C-3E) recovers by expression of a mutant CFL1 resistant to miRNA. We first designed three resistant *CFL1*s (*resCFL1*s) that had seven or eight point mutations, which did not change the amino acid sequences, within the miR-CFL1_1 target sequence (21 bp) (Fig. 4A). Impaired *CFL1* expression by miR-CFL1_1 was indeed rescued by *resCFL1* expression *in vitro*, and the efficiency of expression recovery was highest in resCFL1_1 (resCFL1_1, 36.3 ± 3.4%; resCFL1_2, 32.8 ± 1.7%; resCFL1_3, 28.4 ± 2.8%; *n* = 4: resCFL1_1, *p* = 5.8 × 10^-6^; resCFL1_2, *p* = 2.2 × 10^-5^; resCFL1_3, *p* = 1.3 × 10^-4^ versus miR-CFL1_1 group, Tukey-Kramer’s multiple comparison test) (Fig. 4B). Therefore, resCFL1_1 (henceforth “resCFL1”) was selected for *in vivo* experiments. We also confirmed that the expression level of *resCFL1* was not affected by miR-CFL1_1 *in vitro* (*p* = 0.30, *t*-test) (Fig. 4C). We injected the lentivirus which co-expressed resCFL1 and mCherry into the same cortical region (D2 barrel column) where the lentivirus expressing ChR2-eYFP/miR-CFL1_1 was also injected (Fig. 4D and 4E). After inducing EDP by whisker deprivation, the responses of putative ChR2+ neurons were selectively recorded. This procedure assured miR-CFL1 was expressed in the recorded neurons. Co-expression of ChR2-eYFP and mCherry in the infected cortical area was confirmed by histological analysis (Fig. 4F and 4G). We showed that responses to D1 whisker deflections were significantly larger in neurons expressing both miR-CFL1_1 and resCFL1 than in neurons expressing only miR-CFL1 (miR-CFL1+resCFL1 deprived versus miR-CFL1 deprived, *p* = 0.0069, Tukey-Kramer’s multiple comparison test) (Fig. 4H-4J). These data clearly demonstrate that the effects of miR-CFL1 expression on experience-dependent potentiation were not due to non-specific effects of miR-CFL1 on genes other than *CFL1*, but due to an impairment of CFL1 function.

### CFL1 KD Preserves Experience-Dependent Shrinkage of Deprived Whisker Representation

**Fig 5:**
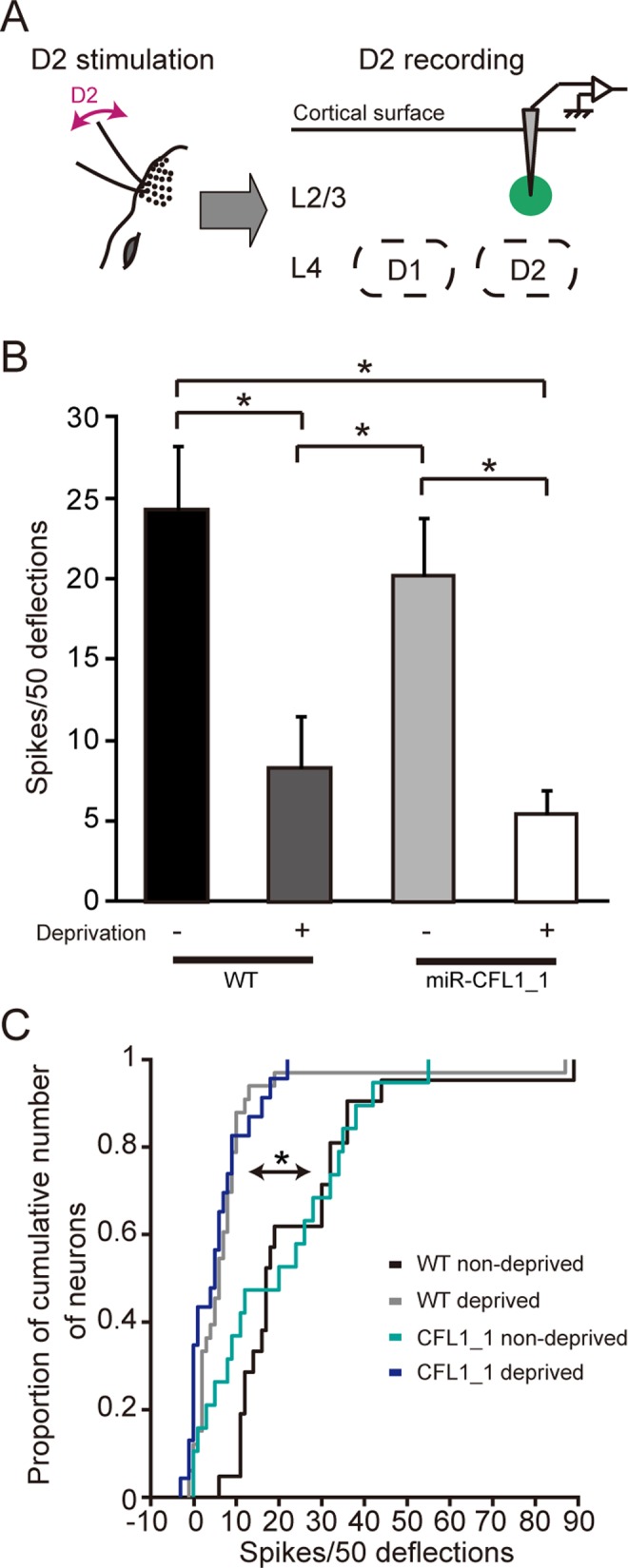
Effects of CFL1 KD on D2 neuronal responses to deprived D2 inputs. (A) Optogenetically labeled CFL1 KD neurons in L2/3 of the D2 barrel column were recorded while the D2 whisker was stimulated. (B) Average number of spikes measured in response to D2 whisker stimulation in D2 column L2/3 neurons for each rat group. *n* = 21, 33, 19, and 23 units for WT non-deprived, WT deprived, miR-CFL1_1 non-deprived, and miR-CFL1_1 deprived groups, respectively. WT non-deprived, *p* = 0.0009; miR-CFL1_1 non-deprived, *p* = 0.028 versus WT deprived group. WT non-deprived, *p* = 0.00025; miR-CFL1_1 non-deprived, *p* = 0.0082 versus miR-CFL1_1 deprived group, Tukey-Kramer’s multiple comparison test. (C) Cumulative frequency histogram of spike number. WT non-deprived, *p* = 5.2 × 10^-8^; miR-CFL1_1 non-deprived, *p* = 0.013 versus WT deprived group. WT non-deprived, *p* = 5.7 × 10^-6^; miR-CFL1_1 non-deprived, *p* = 0.058 versus miR-CFL1_1 deprived group, Kolmogorov-Smirnov test with Bonferroni’s correction.

The same set of cells from which we recorded responses to D1 stimulation was also tested for D2 stimulation (S1 Data). We first confirmed that the L2/3 neurons in the D2 column showed decreased responses to deprived D2 whisker stimulation after sensory deprivation in WT rats (WT non-deprived versus WT deprived, *p* = 0.0009, Tukey-Kramer multiple comparison test) (Fig. 5A-5C). This process is indicative of the shrinkage of deprived whisker input representation (Fig. 1A). We then examined the effects of CFL1 KD on this process, and found that experience-dependent response decrease to the deprived D2 whisker was preserved in either miR-CFL1_1-expressing neurons (WT deprived versus miR-CFL1_1 deprived, *p* = 0.89) (Fig. 5B and 5C) or miR-CFL1_2-expressing neurons (WT deprived versus miR-CFL1_2 deprived, *p* = 0.89) (S3B Fig.). Expression of miR-CFL1_1 in non-deprived rats did not affect L2/3 neuronal responses to D2 stimulation (WT non-deprived versus miR-CFL1_1 non-deprived, *p* = 0.81) (S3B Fig.), suggesting that CFL1 KD itself does not affect L2/3 neuronal responses to whisker stimulation.

Taken together, these results suggest that CFL1-mediated actin dynamics is necessary for the experience-dependent expansion of spared input representation in L2/3, but not for the experience-dependent shrinkage of deprived input representation.

### CFL1-Dependent Increases in Dendritic Spine Number during Functional Barrel Map Plasticity

**Fig 6:**
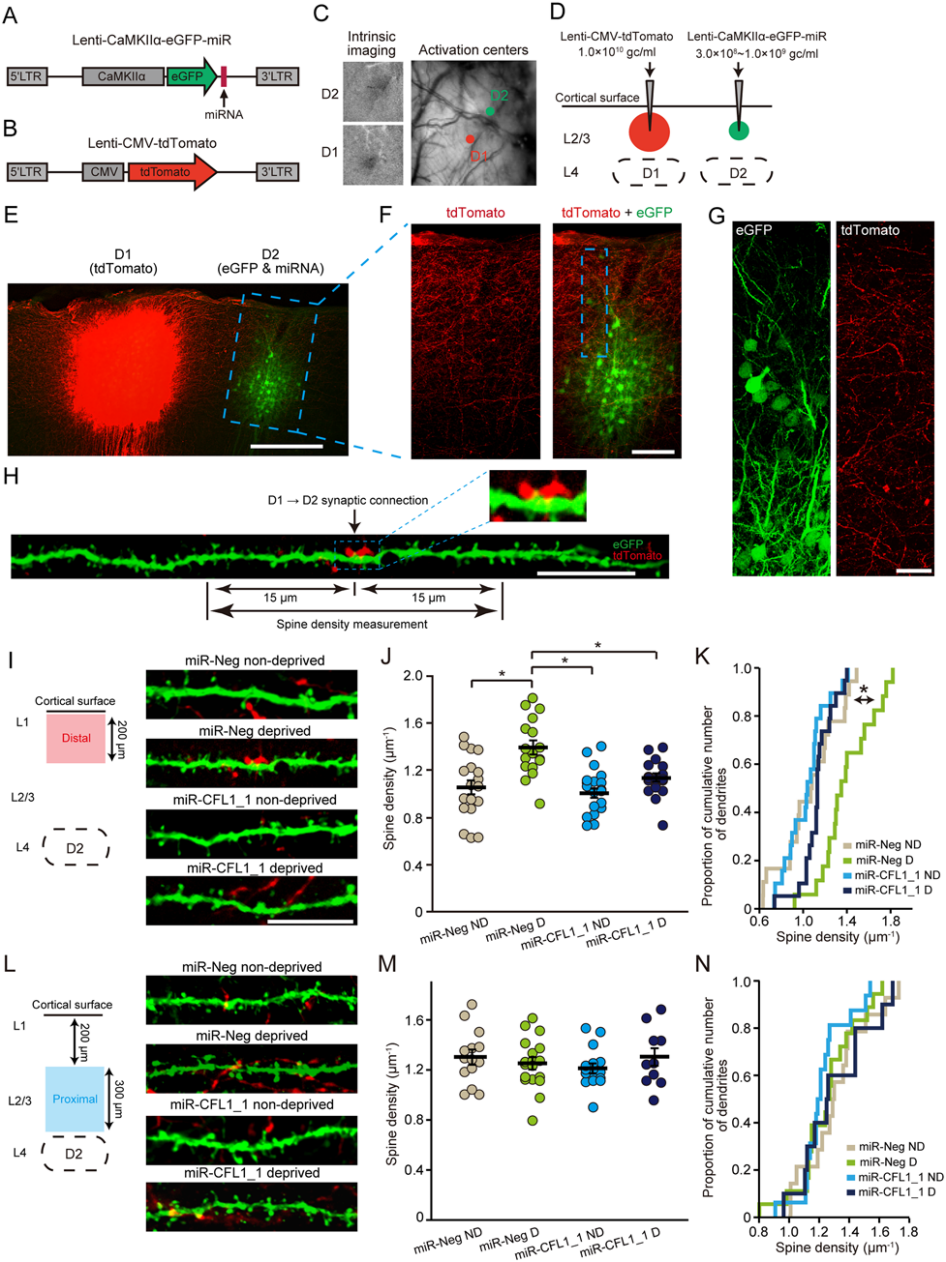
Effects of CFL1 KD on spine density during sensory deprivation. (A, B) Lentiviral vectors employed for co-expressing eGFP and miRNAs (A) and for expressing tdTomato (B). (C) The D1 and D2 barrel columns were identified via intrinsic signal optical imaging. (D) Virus injection was targeted to L2/3 of the D1 and D2 columns. (E) A representative parasagittal section showing expression of tdTomato (D1) and eGFP (D2). Scale bar, 300 μm. (F) Magnified view of an eGFP-expressing region in the parasagittal section shown in (E). Scale bar, 100 μm. (G) Confocal images of a rectangle region shown in (F). Scale bar, 20 μm. (H) A representative dendritic branch in the D2 column is shown that made a putative synaptic connection with a tdTomato+ D1 axonal bouton. Dendritic spines were counted that were localized at a distance less than 15 μm from an identified putative synaptic connection. A magnified view of the putative synaptic connection is shown in the inset. Scale bar, 10 μm. (I) Representative images of the dendritic branches within the distal portion in the D2 column. Scale bar, 10 μm. (J) Spine densities measured in the distal portion. *n* = 18, 17, 19, and 19 branch segments for miR-Neg non-deprived (ND), miR-Neg deprived (D), miR-CFL1_1 ND, and miR-CFL1_1 D groups, respectively. WT ND, *p* = 1.1 × 10^-4^; miR-CFL1_1 ND, *p* = 1.8 × 10^-5^; miR-CFL1_1 D, *p* = 0.0027 versus WT D group, Tukey-Kramer’s multiple comparison test. (K) Cumulative frequency histogram of spine density. WT ND, *p* = 0.037; miR-CFL1_1 ND, *p* = 3.6 × 10^-4^; miR-CFL1_1 D, *p* = 3.6 × 10^-4^ versus WT D group, Kolmogorov-Smirnov test with Bonferroni’s correction. (L−N) Same as (I−K) but of dendritic branches measured within the proximal portion. *n* = 14, 18, 16, and 10 branch segments for miR-Neg ND, miR-Neg D, miR-CFL1_1 ND, and miR-CFL1_1 D groups, respectively. WT ND, *p* = 0.86; miR-CFL1_1 ND, *p* = 0.97; miR-CFL1_1 D, *p* = 0.93 versus WT D group, Tukey-Kramer’s multiple comparison test.

To simultaneously visualize the transcolumnar (D1 → D2) projections and dendritic spines of D2 neurons, we next constructed a new set of lentiviral vectors expressing either tdTomato or enhanced green fluorescent protein (eGFP)/miRNA (Fig. 6A and 6B). Injection of these vector solutions was targeted to the D1 column (tdTomato) or the D2 column (eGFP/miRNA) (Fig. 6C). To avoid dense neuronal expression of eGFP, a low-titer solution of the eGFP/miRNA vector (3.0 × 10^8^ − 1.0 × 10^9^ gc·ml^−1^) was employed (Fig. 6D and 6E). Because the eGFP expression level was low under this low-titer condition and eGFP fluorescence was unendurable for repeated confocal imaging, we used sections stained with an antibody to eGFP for morphological experiments (Fig. 6E). The eGFP/miRNA vector effectively knocked down CFL1 even in the low-titer condition *in vivo* (S4A and S4B Fig.) and in the low multiplicity-of-infection condition *in vitro* (S4C Fig.).

Our findings so far indicate that CFL1 dependency dissociates between two types of barrel map plasticity. Together with the fact that CFL1 is a predominant regulator of dendritic spine structure [29,32], it can be hypothesized that CFL1-mediated structural modifications underlie this dissociation; modification of spine structure occurs selectively in cortical regions where horizontal transcolumnar axons emanating from the spared barrel column exist densely. We thus performed the step-by-step test of this hypothesis.

We first found that the tdTomato intensity emanating from the D1 axons peaked at 150–200 μm from the cortical surface in the D2 column, and decreased with cortical depth (Figs. 6F, 6G, and S5A–S5C). This was in clear contrast to the vertical profile of L4 input strength [44], which was fairly weak at shallow depths and stronger at deeper zones, forming a complementary pattern with that of the D1 axonal intensity profile (S5C Fig.). There was a significant difference in tdTomato intensity between the distal (0–200 μm from the cortical surface) and proximal (200–500 μm) portion (*p* = 0.022, paired *t*-test) (S5D Fig.). These results suggest that a greater number of transcolumnar synaptic connections from the D1 column are generated in the distal portion of the D2 column than in the proximal portion at which the ascending deprived axonal inputs from L4 are thought to predominate.

We next tested the effects of sensory deprivation on dendritic spine number in the miR-Neg (non-deprived and deprived) groups. Learning/experience-driven dendritic spine formation and synaptic plasticity spatially cluster on dendritic branches in cortical pyramidal neurons [45,46] as well as hippocampal neurons [47], and thus spine densities were measured in dendritic branch segments that are supposed to receive dense transcolumnar inputs. For this purpose, we measured spines around (<15 μm) the identified putative transcolumnar connections (Fig. 6H). In non-deprived rats, spine densities were relatively low at the cortical region just below the surface and increased with depth, while densities were nearly constant throughout the supragranular layer in deprived rats (comparison of slopes of regression lines, miR-Neg non-deprived versus deprived, *F*_*1*,*63*_ = 9.33, *p* = 0.0033, *F*-test) (S5E Fig.).

**Fig 7:**
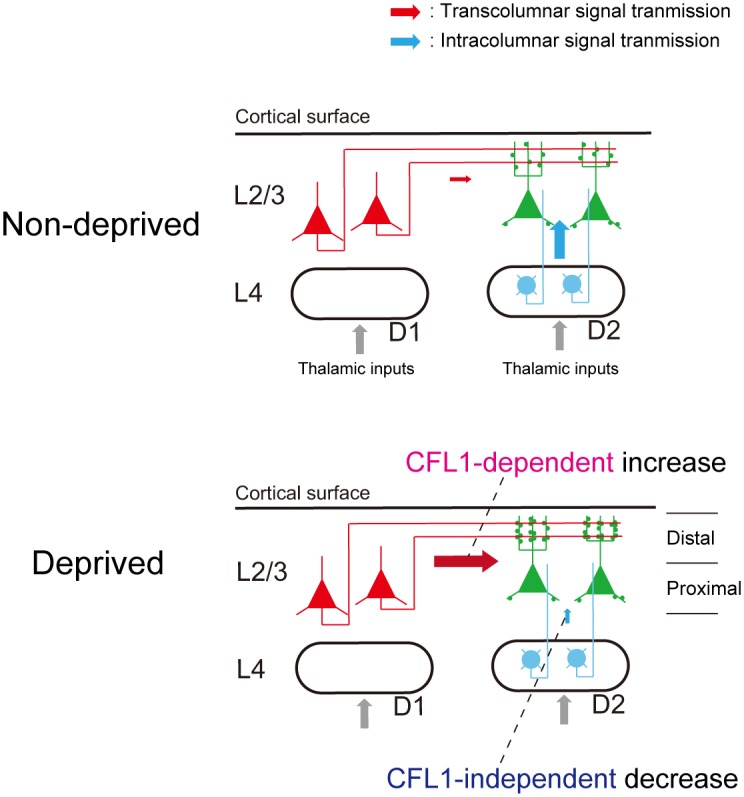
Schematic illustration of the circuit-specific cofilin action on barrel map plasticity. Cross-sectional view of the D1 and D2 barrel columns. L2/3 neurons in the D2 column exhibit a CFL1-dependent neuronal response increase to horizontal transcolumnar inputs from the spared D1 column. By contrast, the response decrease to ascending intracolumnar inputs from L4 is CFL1 independent. In the distal portion of the supragranular layer of the D2 column, spine densities of dendrites receiving transcolumnar inputs from the D1 column increase in a CFL1-dependent manner during sensory deprivation.

These results demonstrate that sensory deprivation affects dendritic spine numbers in a cortical depth-dependent manner. We thus separately compiled dendritic spine densities measured at distal and proximal portions of the D2 column supragranular layer, and examined the impact of CFL1 KD on these values. In the distal portion, spine densities significantly increased with sensory deprivation in the control miR-Neg-expressing neurons (miR-Neg non-deprived versus miR-Neg deprived, *p* = 1.1 × 10^-4^, Tukey-Kramer’s multiple comparison test), but this increase was impaired in the CFL1 KD neurons (miR-Neg deprived versus miR-CFL1_1 deprived, *p* = 0.0027) (Fig. 6I-6K). MiR-CFL1 expression under non-deprived condition did not affect baseline spine densities (miR-Neg non-deprived versus miR-CFL1_1 non-deprived, *p* = 0.97; miR-CFL1_1 deprived versus miR-CFL1_1 non-deprived, *p* = 0.46), suggesting that the effects of CFL1 KD are specific for the deprivation and that the absence of an increase in spine density in miR-CFL1_1 deprived rats was not due to a general reduction in spine density caused by miR-CFL1_1 expression. In contrast, sensory deprivation did not affect dendritic spine densities in the proximal portion of the supragranular layer (miR-Neg non-deprived versus miR-Neg deprived, *p* = 0.34; Fig. 6L-6N). Furthermore, the same conclusion was reproduced even if dendritic spine densities were measured within 5 μm around transcolumnar connections (distal; 1.06 ± 0.07, 1.44 ± 0.07, 1.06 ± 0.06, and 1.09 ± 0.05 spines·μm^−1^ for miR-Neg non-deprived, miR-Neg deprived, miR-CFL1_1 non-deprived, and miR-CFL1_1 deprived groups, respectively; mean ± standard error of the mean [SEM]; miR-Neg non-deprived, *p* = 0.00056; miR-CFL1_1 non-deprived, *p* = 0.0005; miR-CFL1_1 deprived, *p* = 0.0015; versus miR-Neg deprived, Tukey-Kramer’s multiple comparison test) (proximal; 1.18 ± 0.08, 1.31 ± 0.07, 1.24 ± 0.06, and 1.21 ± 0.07 spines·μm^−1^ for Neg non-deprived, Neg deprived, CFL1_1 non-deprived, and CFL1_1 deprived). These observations suggest that during sensory deprivation, CFL1-mediated actin dynamics causally regulate the spine numbers around dendritic spines receiving horizontal transcolumnar synaptic inputs from the spared D1 column. Moreover, these events take place in the distal portion of the D2 column supragranular layer, where dense horizontal transcolumnar projections reside (Fig. 7).

In addition to the dendritic spine density, we also analyzed the sizes of the D2 spines that made putative synaptic connections with horizontally projecting D1 axons. Sensory deprivation did not influence spine sizes in miR-Neg-expressing neurons in either the distal or the proximal portion (miR-Neg ND versus miR-Neg D; distal, *p* = 0.99; proximal, *p* = 0.97; Tukey-Kramer’s multiple comparison test) (S6A and S6B Fig.). In agreement with a previous observation regarding the hippocampal neurons of CFL1 knockout mice [31], spine sizes increased in CFL1 KD neurons in the distal portion (*F*_*1*, *181*_ = 10.7, *p* = 0.0013, main effect of factor 1; factor 1, miR type; factor 2, deprivation type; two-way ANOVA) (S6A and S6B Fig.). However, this increase did not correlate with the neuronal response changes described above (Fig. 3D and 3E), suggesting that the observed spine size changes might not contribute to the functional barrel map plasticity induced by sensory deprivation.

## Discussion

In the present study, CFL1 was locally knocked down in L2/3 excitatory neurons of a deprived column (D2 column) using a lentiviral vector-based RNAi approach. In rats injected with miR-CFL1-expressing vectors, the experience-dependent expansion of the spared input representation was prevented in CFL1 KD neurons, whereas the shrinkage of the deprived input representation was preserved. Furthermore, the spine density around the dendritic spines receiving transcolumnar axonal projections from the spared D1 column was increased in L2/3 neurons of the deprived D2 column, and this increase was impaired by CFL1 KD. These results provide, to the best of our knowledge, the first direct evidence that CFL1-mediated actin dynamics are necessary for plasticity in horizontal transcolumnar circuits during adult cortical EDP.

In rodents, the ADF/cofilin family consists of three genes, namely, *ADF*, *CFL1*, and *cofilin2*. Among their gene products, only ADF and CFL1 proteins are found within the neuronal dendritic spine [21,31]. Importantly, *CFL1* knockout mice exhibit impaired synaptic plasticity at hippocampal synapses [31], while *ADF* knockout mice do not show such deficits [48]. The current investigation therefore focused on *CFL1*. In the present study, we validated the specificity of the effects of CFL1 KD based on three different lines of evidence. Firstly, we showed that miR-CFL1 expression did not affect the expression levels of genes related to *CFL1* including *ADF* (Figs. 2J-2L and S2C). Secondly, we showed that the effect of CFL1 KD was consistent between the two miR-CFL1s with different target sequences within the *CFL1* gene (Figs. 3D and S3A). Because miRNAs can downregulate genes bearing sequences complementary to their seed sequences (positions approximately 2–7 of the guide strand) [43], evidence of the same outcome with different RNAi sequences reduces concerns about the target specificity of RNAi experiments [43,49]. Finally, we showed that impairments of EDP caused by miR-CFL1_1 could be rescued by expression of resCFL1 (Fig. 4). Therefore, our results suggest that the effects observed in the CFL1 KD rats were not due to off-target actions of the miRNAs.

The effects of CFL1 KD on experience-dependent increase of responses to D1 deflections were larger in putative ChR2+ neurons than those in light-responsive putative ChR2—neurons (S2L Fig.). This fact confirmed that experience-dependent potentiation to D1 deflections was impaired in miR-CFL1-expressing D2 neurons. We also showed that responses of light-responsive ChR2—neurons were significantly lower than neurons in WT deprived rats (*p* = 0.0063, *t-*test) (S2M Fig.). This fact suggests that the potentiation of putative ChR2—neurons was also slightly impaired. There are two possible explanations for this observation (which are not mutually exclusive): (1) false negative categorization of ChR2+ neuron as ChR2—neurons by our criteria, and (2) an indirect decrease of responses within the D2 column resulting from a response decrease in ChR2+ neurons surrounding (and potentially connected with) light-responsive ChR2—neurons.

MiR-CFL1 expression did not affect spine density in the proximal part of the L2/3 where ascending axons from L4 dominate, whereas it impaired experience-dependent increase in densities in the distal part where transcolumnar axons dominate (Fig. 6I-6N). In addition to these facts, it is also important to note that CFL1 KD itself does not affect basal spine density in non-deprived rats expressing miR-CFL1 (Fig. 6I-6N). These data suggest that effects of CFL1 KD were specific for dendritic branches receiving “potentiated” transcolumnar inputs.

We found that sensory deprivation increases dendritic spine density selectively in a part of the supragranular layer where dense transcolumnar projections were observed (Fig. 6I-6K). This finding is consistent with those from previous studies showing that sensory deprivation promotes the formation of stable dendritic spines in a deprived column located adjacent to a spared column [20] and also increases the density of the horizontal projections from a spared column to the adjacent deprived columns [50]. By contrast, the absence of dendritic spine structural plasticity in the proximal portion of the D2 column supragranular layer (Figs. 6L-6N and S6B) is consistent with the idea that CFL1-mediated actin dynamics do not involve a decrease in deprived input representation because intracolumnar deprived inputs from L4 are thought to prevail in the proximal portion (S5C Fig.) [15,44]. Taken together, our results suggest that dendritic spines receiving horizontal transcolumnar inputs from the spared column are selectively generated, whereas those receiving ascending intracolumnar inputs from L4 remain constant during sensory deprivation.

The previous report demonstrated that ADF could compensate CFL1 function in the presynapse but not in the postsynapse [51]. Therefore, the absence in functional compensation in deprived rats expressing miR-CFL1 (Fig. 3D and 3E) may suggest that the experience-dependent potentiation in transcolumnar circuits is, at least in part, postsynaptic origin. Interestingly, CFL1 is under the control of calcineurin, a regulator of LTD, and is necessary for the dendritic spine shrinkage associated with hippocampal LTD [28]. Given that LTD contributes to the response decrease to deprived inputs in the barrel cortex [19], it is expected that CFL1 also participates in this process. In hippocampal neurons, however, it is also known that spine shrinkage and the decrease in synaptic transmission efficacy during LTD are dissociated processes at the level of molecular pathways [28]. The present data together with these previous observations suggest that the functional depression of the deprived input representation is likewise dissociated from the CFL1-dependent structural changes in the dendritic spines in the rat barrel cortex. However, this view is inconsistent with the observation reported by Rust and colleagues that postnatal CFL1 knockout impairs hippocampal LTD as well as LTP in mice [31]. Although the reason for this inconsistency is unclear, the function of CFL1 in the experience-dependent depression of the deprived input representation in adult cortex may differ from that in stimulus-induced LTD in hippocampal slices.

We labeled the D1 and D2 columns simultaneously (Fig. 6E). The tdTomato-positive structure observed within the D2 column mostly consisted of axons (Fig. 6G), which validates that our measurements for tdTomato intensity (S5A–S5D Fig.) did not include D1 neuron dendrites travelled from the neighboring column and also that tdTomato signals within the D2 column were not derived from ectopic tdTomato expression outside the D1 column caused by a horizontal spillover of the injection. We accomplished the localized cortical labeling by utilizing the property of lentiviral vectors that have relatively large particle size (~100 nm; [52]) and are thus restricted in its diffusion *in vivo* compared to other vectors such as adeno-associated viral vectors [53]. This labeling technique allowed the demonstration of CFL1-dependent changes in dendritic spine density that could not have been previously accomplished (Fig. 6I-6K). In this analysis, we measured dendritic spines only around the identified putative transcolumnar connections (Fig. 6H). Because tdTomato-expressing area was almost limited within the L2/3 of the D1 column (S7 Fig.), the putative synaptic connections that we identified should consist of monosynaptic connections between D1 L2/3 neurons and D2 L2/3 neurons. On the other hand, it is possible that polysynaptic connections from D1 L2/3 to D2 L2/3 (e.g., D1 L2/3 → D1 L5 → D2 L2/3 [54]) could also have contributed to the dendritic spines that increased around the detected connection, although they are considered to be relatively weak due to the low efficacy of L2/3 → L5 connections [55]. With regard to changes in numbers of synaptic connections, we found it difficult to measure it because the number of D1 neurons labeled with tdTomato (and thus the number of D1 axons that could be detected on D2 dendrites) varied between animals in our small-volume (200 nl) injection method. Indeed, 2-D measurements of the infected areas (on histological sections) in all animals used for spine morphological analysis showed that the sizes of tdTomato-expressing areas varied from the entire supragranular layer of D1 column (~500 μm × 500 μm) to a portion of it (S7 Fig.). This variability in labeled D1 neuron numbers was unavoidable to limit tdTomato labeling within D1 column, and it directly influenced the numbers of synaptic connections that could be detected by this labeling method. It is important to note that it did not affect the spine morphological measurements that were confined to D2 spines connected with D1 axons and the spines around them. Therefore, our strategy may be currently optimal for examining structural modifications in transcolumnar circuits while maintaining between-animal variance at a minimum. However, alternative approaches (e.g., within-animal chronic monitoring of dendritic spines [56]) may also reveal similar conclusions and will be the focus of future studies.

Spine areas measured in the L2/3 distal portion of miR-CFL1 ND and miR-CFL1 D groups showed similar tendency to increase compared to those measured in miR-Neg groups (S6A Fig.). This fact indicates that, regardless of animal’s sensory experience, CFL1 KD itself slightly expands spine sizes. The slight spine expansion was also observed in the hippocampal neurons of CFL1-knockout mice reared under normal environment [31]. Therefore, it is suggested that blocking or decreasing CFL1 action expands dendritic spine sizes independently of each spine’s involvement in sensory EDP. This finding is consistent with the typical role of cofilin as an actin-depolymerizing factor [26]. On the other hand, CFL1 action also promotes actin polymerization [57] and active turnover of actin filaments [29]. Considering these factors together, KD-mediated decrease in CFL1 activity might make dendritic spines more “fixed” state with decreased turnovers and slightly increased sizes. Because active spine turnover is involved in cortical EDP [20,58], our results might suggest that CFL1 regulates EDP by controlling the turnover rates of dendritic spines/actin filaments.

In conclusion, we demonstrate here that CFL1-mediated actin dynamics function in a horizontal connection-specific manner during EDP induced by the single whisker experience protocol. In addition to the rodent vibrissal system, the primate brain is characterized by a highly sophisticated neocortical columnar organization [2]. Cortical circuit reorganization across functional columns through horizontal connections is necessary for functional cortical recovery after peripheral deficits, such as focal retinal lesions [5]. Moreover, plasticity in horizontal transcolumnar or interareal connections is also critical for learning [5]; thus CFL1-mediated circuit reorganization may possibly be a general mechanism for the flexible nature of the human brain. However, this proposal will require further study.

## Materials and Methods

### Ethics Statement

All procedures were performed in accordance with a protocol approved by the University of Tokyo Animal Care Committee (permit number, MED: P11–050). Surgical procedures for lentiviral injection were performed with isoflurane induction (3%) and under maintenance with isoflurane (1%) or ketamine/xylazine (90 mg·kg^−1^ and 10 mg·kg^−1^, respectively) anesthesia. Surgical procedures for electrophysiology experiments were performed under ethyl carbamate (1.2 g·kg^−1^) anesthesia. All efforts were made to minimize suffering and the number of animals employed. Fifty-four male Wistar rats (Nihon SLC) were used for the study (ten were used as WT and 44 were used for viral injection).

### *In Vitro* KD Experiments

Oligonucleotides encoding miRNAs that target the *CFL1* gene were designed with BLOCK-iT Pol II miR RNAi expression vector kits and the associated software (Invitrogen). miR-CFL1_1, miR-CFL1_2, and a negative control miRNA (miR-Neg), which is predicted to not target any known vertebrate gene, were purchased from Invitrogen. miR-CFL1_1 and miR-CFL1_2 target two different regions within the *CFL1* gene (target sequences: miR-CFL1_1, 5′-AGGAATCAAGCACGAATTACA-3′; miR-CFL1_2, 5′-GTTCGCAAGTCTTCAACGCCA-3′).

HEK293T cells (for miRNA screening) or rat PC-12 cells (for examining effects on endogenous gene expression) were used for the *in vitro* experiments. For miRNA screening (Fig. 2A), plasmid vectors expressing miR-CFL1 (pcDNA6.2-GW/EmGFP-miR vector; Invitrogen) and the rat *CFL1* gene (pCAG-rCFL1, the kind gift of H. Kasai, University of Tokyo) were co-transfected into the HEK293T cells. Three days after transfection, total RNA was prepared and used as a template for real-time reverse-transcriptase PCR with the *StepOne* Real-Time PCR system (Applied Biosystems).

For resCFL1 screening (Fig. 4B), pcDNA6.2-GW/EmGFP-miR-CFL1, pCAG-rCFL1, and the plasmid vector expressing *resCFL1* (pCMV-resCFL1) were co-transfected. For generation of the pCMV-resCFL1, PCR fragments encoding each of the N-terminal and C-terminal side of *CFL1* and an annealed oligonucleotide encoding mutated portion of *CFL1* (resCFL1_1, 5′-CAAGAAGAAACTGACTGGCATTAAACATGAGCTCCAAGCTAACTGCTACGA-3′; resCFL1_2, 5′-CAAGAAGAAACTGACGGGTATCAAACATGAGCTCCAAGCTAACTGCTACGA-3′; resCFL1_3, 5′-CAAGAAGAAACTGACCGGGATAAAACATGAGCTCCAAGCTAACTGCTACGA-3′) were simultaneously fused to *EcoR*I/*Not*I-digested pIRES2-AcGFP1 (Clontech) by using InFusion Cloning kit (Clontech). Primer sequences were as follows: CFL1-N-F, 5′-CTCAAGCTTCGAATTACCGGTATGGCCTCTGGTGTGGCT-3′; CFL1-N-R, 5′-GTCAGTTTCTTCTTGATGGCATCC-3′; CFL1-C-F, 5′-AGCTAACTGCTACGAGGAGGTCAA-3′; CFL1-C-R, 5′-TCTAGAGTCGCGGCCGCTCACAAAGGCTTGCCCTC-3′;

To examine the effects of miR-CFL1 expression on endogenous gene expression (Figs. 2B, 2J, 2K, S1A, and S1C), the PC-12 cells (1 × 10^5^) were transfected with the Lenti-CMV-hChR2-eYFP-miR-CFL1_1 or-miR-CFL1_2 vector (2.0 × 10^7^ gc). Four days later, total RNA was prepared from these cells or the cells were solubilized and the cell extracts were obtained. For protein expression analysis, the extracts were immunoblotted [59] using the following antibody combinations: rabbit antibody to cofilin (1:250; Cytoskeleton) or to destrin (1:1,000; Sigma-Aldrich)/horseradish peroxidase-conjugated antibody to rabbit IgG (1:2,000; Rockland Immunochemicals Inc.), and mouse antibody to β-actin (1:5,000; Sigma-Aldrich)/horseradish peroxidase-conjugated antibody to mouse IgG (1:1,000; Vector Laboratories, Inc.). The band intensities were quantified by using ImageJ software (National Institutes of Health).

The primer sets used for reverse-transcriptase PCR experiments were as follows: CFL1-F, 5′- GCTCTTTTGCCTGAGTGAGG-3′; CFL1-R, 5′-CTTAAGGGGTGCACTCTCG-3′; ADF-F, 5′-GTGCATAGTCGTTGAAGAAGG-3′; ADF-R, 5′-CCTTCGAGCTTGCATAGATC-3′; Twf1-F, 5′-CTGAGTAAGAGACAGCTCAACTATG-3′; Twf1-R, 5′-GCTCTCTTATGCTGCATGTG-3′; Twf2-F, 5′-CTGAAGATGCTGTATGCAGC-3′; Twf2-R, 5′-CTGGTGCTTACTCTCCACAC-3′; GAPDH-F, 5′-TGAACGGGAAGCTCACTGG-3′; GAPDH-R, 5′-TCCACCACCCTGTTGCTGTA-3′.

### Lentiviral Transfer Vector Construction and Lentiviral Preparation

For generation of the lentiviral transfer vector, pCL20c CaMKIIα-hChR2-eYFP-miR, the *Pac*I/*Bam*HI-digested mouse CaMKIIα promoter (1.3 kb) from pLenti-CaMKIIα-hChR2-mCherry-WPRE (the kind gift of K. Deisseroth) was inserted into *Mlu*I/*Eco*RI-digested pCL20c MSCV-hChR2-eYFP [37] by blunt-end ligation to replace the MSCV promoter. A PCR fragment encoding miR-CFL1 or miR-Neg was inserted into the *Cla*I site of pCL20c CaMKIIα-hChR2-EYFP using an InFusion Cloning kit. For generation of pCL20c CaMKIIα-eGFP-miR, a PCR fragment encoding miRNA was inserted into the *Cla*I site of pCL20c CaMKIIα-eGFP [39]. For generation of pCL20c CaMKIIα-mCherry-P2A-resCFL1, PCR fragments encoding each of *mCherry* (derived from pmCherry-N1 vector, Clontech) and *resCFL1* were simultaneously fused to *Age*I/*Not*I-digested pCL20c CaMKIIα-eGFP by using InFusion kit. The P2A sequence was separately added to each of the primers used to amplify *mCherry* and *resCFL1* (primer sequences: mCherry-F, 5′-CCCGGGATCCACCGGCGCCACCATGGTGAGCAA-3′; mCherry-P2A-R, 5′-CTGCTTGCTTTAACAGAGAGAAGTTCGTGGCTCCGGAGCCCTTGTACAGCTCGTCCATGCC-3′; P2A-resCFL1-F; 5′-TGTTAAAGCAAGCAGGAGACGTGGAAGAAAACCCCGGTCCCATGGCCTCTGGTGTGGCTGTC-3′; resCFL1-R, 5′-ATTATCGATGCGGCCTCACAAAGGCTTGCCCTC-3′). The lentiviral vectors were produced and titrated by using the DNA titration method, as described previously [35].

### Whisker Trimming and Stimulation

All whiskers, except for the D1 whisker, on the left side of the face were trimmed by cutting the whiskers to fur level (<1 mm) under brief isoflurane anesthesia (3%) by using an anesthetizer (MK-AT200D; Muromachi Kikai). The ipsilateral whiskers were not trimmed. Rats were 10 weeks old at the onset of whisker trimming. Subsequently, the whiskers were re-trimmed every 2 days. Trimming was continued for more than 3 weeks (range 23–45 days) and ceased at 1 week before electrophysiological recordings to stimulate the regrowth of the trimmed whiskers.

Each whisker was inserted into a glass capillary (inner diameter, 0.5 mm) glued to a piezoelectric bending element. A stereoscope was used to insert the whisker to a distance of 10 mm from the whisker pad. For electrophysiological recording, 200 μm ventral-dorsal deflections (10 ms at 1 Hz, repeated 50 times) were applied, resulting in an angular deflection of 1.14°. For intrinsic signal optical imaging, 1–2° amplitude ventral-dorsal deflections (50 ms at 10 Hz, repeated 50 times) were applied.

### Intrinsic Signal-Based Lentiviral Injection

Rats were 8 weeks old at the time of lentiviral vector injection. Anesthesia was induced with 3% isoflurane and anesthesia was maintained by either 1% isoflurane, ketamine (90 mg∕kg IP)∕xylazine (10 mg∕kg IP). Each rat was positioned in a stereotaxic apparatus (SR-6R; Narishige). The skull over the barrel cortex was carefully thinned to create a cranial window.

Functional maps of the barrel cortex were determined by using intrinsic signal optical imaging. The cortical surface was illuminated with a red light (wavelength, 705 nm) while stimulating a single whisker. Images were collected with a charge-coupled device (CCD) camera (Tokyo Electric Industry) and digitized with an IBM/PC-compatible video system equipped with a video frame grabber board (Matrox Imaging). The imaged area was a 4.2 × 3.1 mm region with a spatial resolution of 320 × 240 pixels. The surface blood vessels were imaged by using a green light (wavelength, 540 nm). The focusing depth was adjusted to 500 μm below the cortical surface. For each recording trial, data were collected for 8 s with a frame length of 0.5 s (16 frames per trial). Reflectance changes in response to whisker stimulation were estimated by subtracting a 3 s averaged frame taken before the onset of whisker stimulation from a 3 s averaged frame taken at the time of whisker stimulation.

To prepare the rats for the electrophysiology experiments, the D2 barrel column was first identified, and a glass pipette (tip diameter, ~40 μm) (sharply [>45°] grinded so as to make depth control easier and to mitigate the spillover of cerebrospinal fluid) attached to a 1 μl Neuros syringe (7001 KH; Hamilton Company) was then vertically inserted into the center of the D2 barrel column to a depth of 300 μm from the cortical surface. Before the pipette insertion, a mannitol solution (25% in saline) was intraperitoneally injected to mitigate the spillover of cerebrospinal fluid. Next, a solution of the lentiviral vector (Lenti-CaMKIIα-hChR2-eYFP-miR vector containing miR-CFL1_1, miR-CFL1_2, or miR-Neg; 200 nl of 1.0 × 10^10^ gc·ml^−1^ solution; *n* = 10, 5, and 6 rats, respectively) or a mixed solution (1:1) of two vectors (Lenti-CaMKIIα-hChR2-eYFP-miR-CFL1_1 [1.0 × 10^10^ gc·ml^−1^] and Lenti-CaMKIIα-mCherry-P2A-resCFL1 [1.0 × 10^10^ gc·ml^−1^], 200 nl, *n* = 4 rats) was injected at a flow rate of 25–50 nl·min^−1^ with the aid of a micropump (UltramicroPump III; World Precision Instruments [WPI]) and a microprocessor-based controller (Micro4; WPI). The needle was left in place for additional 15 min. The scalp incision was carefully sutured, and the rat was returned to a standard cage after recovering from anesthesia.

To prepare the rats for the morphology experiments, both D1 and D2 barrel columns were functionally identified prior to lentiviral vector injection. Solutions containing either the Lenti-CMV-tdTomato-WPRE vector (200 nl; 1.0 × 10^10^ gc·ml^−1^) or the Lenti-CaMKIIα-eGFP-miR vector (200 nl; 3.0 × 10^8^ − 1.0 × 10^9^ gc·ml^−1^; miR-CFL1_1, *n* = 7 rats; miR-Neg, *n* = 7 rats) were then injected into the center of the D1 or D2 barrel column, respectively.

### Laser Optical Stimulation

A fiber enclosed in the glass coated optrode was coupled to a diode laser (peak wavelength at 473 nm, Omicron Laserage Laserprodukte GmbH). The timing of the stimulation was managed with an electrically controlled mechanical shutter (UNIBLITZ). The light power was controlled with a Neutral Density (ND) filter (Thorlabs). For each neuron, 10 × 10 light pulse trains, each with a duration of 5 ms, were delivered at 1 and 20 Hz. The interval between each train was 15 s [37]. The light intensity was adjusted after observing the neuronal responses so as to avoid the skew of light-evoked spike waveforms from spontaneous spike waveforms [36]. More specifically, the maximum light intensity that did not skew waveforms under visual inspection was employed. The light power at fiber input was in the range of 0.1 − 5 mW.

### *In Vivo* Electrophysiological Recording

Each rat was anesthetized with ethyl carbamate (1.2 g·kg^−1^). The body temperature was maintained at 37.5°C throughout the experiment. A catheter (Natsume Seisakusho) was surgically inserted into the left femoral vein [35]. Ringer’s solution and additional doses of anesthesia (urethane, 0.2 − 0.4 g·kg^−1^) were then administered through the catheter. The skull over the barrel cortex was exposed and carefully removed. *In vivo* eYFP fluorescence was identified by using a cooled CCD camera (VB-7000; Keyence) attached to a fluorescence stereoscopic microscope (VB-G05; Keyence).

The activities of single neurons were extracellularly recorded using a glass-coated tungsten microelectrode (impedance < 1 MΩ) in WT rats, or a glass-coated tungsten optrode (impedance < 1 MΩ) [37] in ChR2-expressing rats. The electrode was vertically inserted into the cortex via a hydraulic micromanipulator (MO-10; Narishige). Neuronal signals were amplified with an AB651J amplifier (Nihon Kohden), band-pass filtered (0.4–5 kHz filter; Nihon Kohden), digitized at 25 kHz, and stored by using the Recorder Software (Neural Data Acquisition System). Single units were obtained in the off-line analysis with Offline Sorter Software (Plexon) [60,61]. Briefly, the SD was first calculated to estimate the variance of the baseline noise. Spikes were then extracted using a threshold of >5 × SD from the baseline mean. The information encoded in spike waveforms was compressed using principal component analysis. If waveforms with shapes uncharacteristic of neuronal action potentials were existed, they were excluded before the calculation of principal components. A cluster was selected in 2-D or 3-D feature (typically using the first three principal components) space by drawing a contour manually. The presence of a refractory period was confirmed in the autocorrelogram. If the number of spikes with interspike intervals < 2 ms exceeded 1% of the total for a given unit, the unit was discarded or additional feature combinations were examined to subdivide the cluster further until meeting the criteria in the autocorrelogram [60,61]. Single-unit data were analyzed with MATLAB software (MathWorks).

Recordings were performed from both L2/3 (depth from the pial surface, 0–500 μm) and L5 (800–1250 μm) [62] neurons. In some cases, electrolytic lesions (1 μA, 5 s, tip negative) were applied at a depth corresponding to L4 (750 μm) to map recording locations onto the barrel pattern.

In parallel with the single-unit recordings, cortical electroencephalograms were also recorded to monitor the cortical state. A stainless steel screw was threaded into the bone above the occipital cortex. For reference, another screw was threaded into the bone above the cerebellum. Signals were amplified with an AB-610J amplifier, band-pass filtered (0.5–100 Hz), and stored. During whisker stimulation trials, anesthesia was maintained to a depth equivalent to stage III slow-wave sleep, as described previously [63].

### Brain Tissue Processing and Cell Counting

Rats were perfused with saline, followed by 4% paraformaldehyde in phosphate buffer. The brains were post-fixed in 4% paraformaldehyde for 2–4 h and immersed in a solution of 20% sucrose in PBS. To recover the location of the electrophysiological recordings and virus expression, the cortex was flattened between two glass slides, sectioned at 50 μm, and processed for cytochrome oxidase staining (2–3 h at room temperature [RT] in 20 ml phosphate buffer containing 10 mg diaminobenzidine [DAB], 10 mg cytochrome c, and 0.8 g sucrose) [64]. Blood vessels were used as a reference for projecting the barrel patterns in L4 onto the eYFP-expressing L2/3 sections.

Although ChR2-eYFP expression is mostly restricted to the cell membrane [65], weak fluorescence was also observed at the cytoplasm (Fig. 1J). No eYFP fluorescence was observed at the cell nuclei. To confirm L2/3-restricted expression of eYFP, we thus stained ChR2-eYFP-expressing brain sections with an antibody against MAP2, which stains neuronal cytoplasm as well as dendrites [66], rather than with an antibody against neuron-specific nuclear protein (NeuN), which mainly stains nuclei [67].

For the detection of MAP2 or NeuN in selected neuronal populations, coronal sections (25 μm thick) were immunostained with either mouse antibody to MAP2 (1:2,000; Sigma-Aldrich) or mouse antibody to NeuN (1:1,000; Millipore). The sections were reacted with an Alexa Fluor 647-conjugated antibody to mouse IgG (1:500; Invitrogen). Sections were counterstained with the DNA-specific fluorescent dye, Hoechst 33342 (Invitrogen).

To visualize expression of CFL1 or ADF, sections were immunostained with a rabbit antibody to cofilin (1:250) or a rabbit antibody to ADF (1:100), followed by either an Alexa Fluor 647-conjugated antibody to rabbit IgG (1:500; Invitrogen) or a horseradish peroxidase-conjugated antibody to rabbit IgG (Dako Corporation) and DAB. The stained images were obtained by using a BZ-9000 fluorescence microscope (Keyence) and a TCS-SPE confocal microscope (Leica).

Confocal images were used for cell counting. For estimating layer distribution of eYFP+ neurons, sections double-stained with MAP2 and Hoechst were used. Layers were manually identified based on differences in cell density and size. Three non-adjacent sections were chosen that encompassed each injection point, and all eYFP+ neurons were counted in each section. For counting the percentages of CFL1+ neurons, CFL1- and NeuN-stained sections were used. Three region-of-interests (ROIs, 183.3 × 183.3 μm) were randomly selected from both eYFP-expressing region and neighboring normal cortical region (where eYFP was not expressed), and all CFL1+ and NeuN+ neurons were counted within each ROI.

### Morphometric Analysis of Spine Density, Spine Area, Axonal Density, and tdTomato-Positive Area

For all morphological analyses, the parasagittal floating sections (50 μm thick) were prepared from the rat cortex that included both the D1 column and the D2 column. Immunohistochemistry was performed as described previously [25]. Briefly, sections were immunostained with chicken antibody to GFP (1:500; Abcam) and rabbit antibody to DsRed (1:500; Clontech), followed by Alexa Fluor 488-conjugated anti-chicken (1:200; Invitrogen) and Alexa Fluor 546-conjugated anti-rabbit (1:200; Invitrogen) secondary antibodies.

For morphometric analysis of dendritic spine density and area on immunostained section [24], confocal immunofluorescence images (voxel size, 0.1 × 0.1 × 0.5 μm^3^) were acquired with a CSU-22 spinning-disc confocal unit (Yokogawa Electric) coupled to an Axiovert 200M microscope through a Plan Apochromat 63× objective (NA 1.4; Carl Zeiss). The acquired images were then analyzed using MetaMorph software (Universal Imaging Corporation). Given sets of tdTomato+ D1 axons and eGFP+ D2 dendritic spines were defined as synaptically connected if both fluorescent signals were found within the same voxel.

The density of the dendritic spines (including all types of spines, e.g., thin, mushroom-shaped, and stubby spines) was measured at dendritic branch segments that met the following criteria: (1) the segment was nearly parallel to the xy plane (1,280 × 1,010 pixels) in a stacked image of consecutive focal planes (typically, 10–50 stacked planes) taken at 0.5 μm intervals in the z direction; (2) at least one putative synaptic connection with a tdTomato+ axon was identified; (3) no bifurcations were present within the segment; (4) the segment demonstrated no crossing with other branches. For identification of putative synaptic connections, we did not consider overlaps between dendritic shaft and axons. Therefore, all putative synapses were identified on the basis of overlaps between dendritic spines and axons. Branch segments in which the total measured length was less than 4 μm were discarded. Spines within 15 μm of the identified putative synaptic connection were counted, along with dendritic length. In some cases (eight of 51 dendrites in the distal portion and five of 41 dendrites in the proximal portion), two or more connections were identified on a single dendritic branch. If distance between a given connection pair was less than 2 × 15 μm (five of eight in distal and four of five in proximal), two connections were regarded as forming a single branch segment. If this was not the case (three of eight dendrites in distal and one of five in proximal), two connections were regarded as forming different branch segments with each other. We also measured spine densities within 5 μm from connections using the same criteria.

For measurement of the dendritic spine area on immunostained section, the fluorescence images were first thresholded, where the threshold was more than the mean plus 5 × the standard deviation (SD) of the background intensity distribution. We did not normalize intensity levels in each image. Spines that made putative synaptic connections with D1 axons were then identified. Next, a 2-D projection image was reconstructed from the z plane that included the identified spine. Spine area was estimated in the 2-D image by manually enclosing the spine head, followed by measurement of the total pixel number included within the enclosure. All types of spine morphologies were included in the analysis, and all measurements of spine density and spine area were performed by investigators who were blind to the animal’s sensory experience and the identity of the injected virus.

For analysis of axonal density on immunostained section, fluorescence images were acquired with a BZ-9000 microscope through a Plan Apochromat 20 × objective (NA 0.75; Nikon). The intensity of the tdTomato fluorescence attributable to D1 column axons was measured within a region of interest (100 × 50 μm^2^) localized within the D2 column where the eGFP signal was observed. The region of interest was vertically scanned from the surface of the cortex to the superior end of L4 (depth, 500 μm). Measurements were performed in three sections for each rat. The background intensity (measured at L4 for each section) was first subtracted from the determined intensity of the tdTomato fluorescence, and then the subtracted intensity was normalized to the value of the baseline-subtracted maximum intensity, and then averaged across the three sections. For measurement of tdTomato-positive area (S7 Fig.), fluorescent images were thresholded at 10× background intensity of each section (measured within L4) and binarized. TdTomato-positive area was estimated by manually enclosing the signal-positive area in the binarized image.

### Electrophysiology Data Analysis

Data analysis was performed with MATLAB software. Light-responsive neurons were identified using 1 Hz stimulation data, by comparing firing rates as a function of stimulation latency during the first 100 ms after each light pulse with the firing rates obtained for similar time blocks after shuffling the spike times of each cell within an interval (−100, +100 ms) around stimulation onset [38]. After each shuffling, spikes were counted in 1 ms bins, and the three successive bins that showed the maximum spike numbers during the 100 ms period after stimulation onset were identified. The spike times were shuffled 10,000 times for each cell. Three successive bins with a maximum spike numbers were also identified for the real data. Cells were classified as light-responsive if the number of spikes in the three-bin block with the maximal spike numbers in the real data exceeded the 99.9th percentile value of the distribution of the maximum spike numbers in the shuffled data. The latency of the response was defined as the mean latency of all spikes contributing to this block.

Light-responsive ChR2− neurons were previously reported as showing a lower spike probability for high frequency repetitive light stimulation compared with ChR2+ neurons [34,36–38]. Therefore, repetitive light pulses (20 Hz) were applied to each neuron to examine the probability of spike. The first light pulse in each 10-pulse train was excluded from the analysis. The number of spikes evoked by 90 light pulses was estimated as the spike number detected during the 25 ms after light onset subtracted by the spike number detected during the 25 ms before light onset. In our experimental preparation, ChR2+ somata were almost completely absent in L5, but ChR2+ axons originating from ChR2+ L2/3 neurons were abundant in L5. Thus, light-responsive L5 neurons were considered to be a pure population of “indirectly” activated neurons. Indeed, neurons that reliably responded to repetitive optical stimulation were observed in L2/3, while the firing probability was lower in L5 (S2D–S2G Fig.), consistent with previous reports; thus putative ChR2+ neurons were defined as those neurons exhibiting a higher reliability than the neuron that showed the highest reliability in L5 (S2F and S2G Fig.).

Recording locations were reconstructed based on the relative location of the lesion marks within the barrel patterns, as visualized by cytochrome oxidase staining in tangential sections. To allocate each recording location within the D2 barrel column, the center of the mass was calculated for D1 and D2 columns. A line passing through the center of both D1 and D2 was drawn, and each recording location was then vertically projected onto the line. Distance of each recording location from the D1 column center was measured and normalized to the distance between D1 and D2 centers. In one out of 32 rats (miR-CFL1_1 deprived rats, seven putative ChR2+ units from four recording tracks), we failed to reconstruct the barrel pattern histologically, and thus estimated column centers and recorded locations based on intrinsic signal optical imaging data.

### Statistics

All statistical tests for the *in vivo* study were performed with MATLAB and freely available R software. Data are given as the means, and error bars denote the standard error of the mean, except when indicated otherwise.

## Supporting Information

S1 Data Excel spreadsheet containing the underlying numerical data for Figs. 1–6 and S1–S7. (XLSX)

S1 Fig CFL1 protein KD in vitro and in vivo. (A) CFL1 protein KD efficiency of miR-CFL1_1 and miR-CFL1_2 in PC-12 cells. CFL1 protein levels were normalized to those of the miR-Neg group. *n* = 3 for all groups. miR-CFL1_1, *p* = 0.0036; miR-CFL1_2, *p* = 0.0073 versus miR-Neg, Dunnett’s multiple comparison test. (B) Two neighboring coronal sections from a miR-CFL1_2 virus-injected rat are shown, one depicting eYFP fluorescence (top) and the other depicting CFL1 immunoreactivity (bottom). (C) Effects of miR-CFL1 on ADF protein expression in PC-12 cells. *n* = 3 for all groups. miR-CFL1_1, *p* = 0.094; miR-CFL1_2, *p* = 0.078: versus miR-Neg.
(TIF)

S2 Fig Classification of recorded units. (A, B) Representative waveforms of a regular-spiking neuron (A) and a fast-spiking neuron (B). Gray lines indicate single spike waveforms, and black lines show averaged waveforms (*n* = 30). Horizontal scale bar, 200 μs; vertical scale bar, 150 μV. (C) Population data. The wave duration (difference between peak and trough time) and peak/trough ratio of the waveform for each neuron are plotted (*n* = 332). The magenta symbols correspond to neurons that were classified as fast-spiking neurons. (D, E) Representative response patterns of L5 (D) and L2/3 neurons (E) to 20 Hz repetitive light stimulation. Scale bars: horizontal, 200 ms; vertical, 200 μV. (F, G) Distribution of spike probability in light-responsive neurons of L5 (F) and L2/3 neurons (G). The dark gray bars in G correspond to neurons that were classified as putative ChR2+ neurons, and the white bars correspond to neurons that were classified as putative ChR2− neurons. The distribution shown in F was the result of unbiased sampling. By contrast, it is important to note that the distribution shown in G was the result of biased sampling; we searched neurons that showed high response reliability to the repetitive light stimulation online. (H−J) Latency distribution of L2/3 putative ChR2+ (H), L2/3 putative ChR2− (I), and L5 light-responsive neurons (J) (*n* = 92 (L2/3 ChR2+), 54 (L2/3 ChR2–), and 34 (L5 light-responsive). (K) Cumulative frequency histogram of latency for neuronal population. None of the combination showed significant difference (L2/3 ChR2+ versus L2/3 ChR2−, *p* = 0.90; L2/3 ChR2+ versus L5 light-responsive, *p* = 0.16; L2/3 ChR2− versus L5 light-responsive, *p* = 0.21; Kolmogorov-Smirnov test). (L) Average number of spikes measured in putative ChR2+ and ChR2− L2/3 neurons in response to D1 whisker stimulation for each rat group is shown. *n* = 23, 28, 18, and 25 units for miR-CFL1_1 ChR2+, miR-CFL1_1 ChR2−, miR-CFL1_2 ChR2+, and miR-CFL1_2 ChR2−, respectively. *F*_*1*, *90*_ = 9.01, *p* = 0.0035, main effect of factor 1; factor 1, neuron type; factor 2, miR type; two-way ANOVA: miR-CFL1_1, *p* = 0.017; miR-CFL1_2, *p* = 0.15; ChR2+ versus ChR2−, *t-*test with Bonferroni’s correction. (M) Comparison of responses to D1 whisker stimulation between regular-spiking (RS) neurons recorded in WT deprived rats and light-responsive ChR2− neurons recorded in deprived rats expressing miR-CFL1. *n* = 33 and 53 units for RS in WT deprived and ChR2− in miR-CFL1 deprived, respectively. *p* = 0.0063, *t*-test.
(TIF)

S3 Fig Effects of miR-CFL1_2 or miR-Neg expression on experience-dependent plasticity in D2 neuronal responses. (A) Average number of spikes measured in D2 neurons in response to D1 whisker stimulation for each rat group is shown. *n* = 33, 18, and 16 units for WT deprived, miR-CFL1_2 deprived, and miR-Neg deprived, respectively. WT deprived versus miR-CFL1_2 deprived, *p* = 4.1 × 10^-6^, Tukey-Kramer’s multiple comparison test. (B) Average number of spikes measured in D2 neurons in response to D2 whisker stimulation for each rat group. *n* = 33 and 18 units for WT deprived and miR-CFL1_2 deprived groups, respectively. *p* = 0.89, Student’s *t*-test.
(TIF)

S4 Fig CFL1 KD via Lenti-CaMKIIα-eGFP-miR-CFL1 vector. (A) Two neighboring coronal sections from a Lenti-CaMKIIα-eGFP-miR-CFL1_1-injected rat (titer, 6.3 × 10^8^ gc·ml^−1^) are shown, one depicting eGFP fluorescence (top) and the other depicting CFL1 immunoreactivity (bottom). Scale bar, 300 μm. (B) Magnified view of the rectangular region indicated in (A). Scale bar, 100 μm. (C) Lentiviral titer dependence of KD efficiencies measured *in vitro*. Rat CFL1-overexpressed HEK293T cells (8.0 × 10^4^ cells) were infected with the miR-CFL1_1 expressing vector (Lenti-CMV-ChR2-eYFP-miR-CFL1_1) in different titer conditions (1.6 × 10^7^, 1.6 × 10^6^, and 8.0 × 10^5^ gc for undiluted, 1/10, and 1/20 groups, respectively). ‘“WT” indicates uninfected cells. *n* = 3 for all groups. Undiluted, *p* = 4.1 × 10^-6^; 1/10, *p* = 3.8 × 10^-6^; 1/20, *p* = 5.2 × 10^-6^ versus WT, Tukey-Kramer’s multiple comparison test.
(TIF)

S5 Fig Division of the supragranular layer into distal and proximal portions. (A) Magnified view of an eGFP-expressing region in the parasagittal section shown in Fig. 6E. Scale bar, 100 μm. (B) The tdTomato fluorescence intensity derived from D1 axons was measured in the rectangular region of interest in the D2 column. The rectangle was vertically scanned within the D2 column. (C) An averaged vertical profile of normalized tdTomato fluorescence intensity (black line). Gray lines indicate the data for each rat (*n* = 4 rats). See Experimental procedures for details regarding the normalization of fluorescence intensity and cortical depth. A vertical profile of L4 input strength measured in Petreanu and colleagues was also plotted on the same graph [44] (blue dashed line). Based on these observations, the supragranular layer was separated into the distal portion (0–200 μm from the cortical surface) and the proximal portion (200–500 μm), in which horizontal transcolumnar inputs or ascending intracolumnar inputs, respectively, are thought to predominate. (D) The tdTomato fluorescence intensity was averaged in either the distal or proximal portion of the supragranular layer. *p = 0.022, paired *t*-test. (E) Dendritic spine densities measured around (<15 μm) spines receiving D1 inputs in the miR-Neg non-deprived (ND) and deprived (D) groups were plotted against the cortical depth of the midpoints of the measured dendritic segments (bin width, 50 μm). Each triangle and circle represents a dendritic branch segment for the miR-Neg ND and D groups, respectively. The dashed and solid lines correspond to lines fitted to the distribution of miR-Neg ND and D groups, respectively, by linear regression. *F*_*1*,*63*_ = 9.33, *p = 0.0033, *F*-test (the null hypothesis stated that the slopes of the lines fitted by linear regression were equivalent).
(TIF)

S6 Fig Spine area analysis. (A) Plots of dendritic spine areas measured in the distal portion of L2/3 for each rat group (left) and cumulative frequency histogram (right). *n* = 48, 41, 40, and 56 spines for miR-Neg ND, miR-Neg D, miR-CFL1_1 ND, and miR-CFL1_1 D, respectively. *F*_*1*, *181*_ = 10.7, *p* = 0.0013, main effect of factor 1; factor 1, miR type; factor 2, deprivation type; two-way ANOVA. miR-Neg ND versus miR-CFL1 ND, *p* = 0.10; miR-Neg ND versus miR-CFL1 D, *p* = 0.060; miR-Neg D versus miR-CFL1 ND, *p* = 0.060; miR-Neg D versus miR-CFL1 D, *p* = 0.033: Tukey-Kramer’s multiple comparison test. miR-Neg ND versus miR-CFL1 ND, *p* = 0.10; miR-Neg ND versus miR-CFL1 D, *p* = 0.11; miR-Neg D versus miR-CFL1 ND, *p* = 0.12; miR-Neg D versus miR-CFL1 D, *p* = 0.038: Kolmogorov-Smirnov test with Bonferroni’s correction. (B) Same as (A) but of dendritic spine areas measured in the proximal portion. *n* = 26, 40, 45, and 38 spines for miR-Neg ND, miR-Neg D, miR-CFL1_1 ND, and miR-CFL1_1 D groups, respectively.
(TIF)

S7 Fig Variability in sizes of tdTomato-positive areas across rats. (A) Example coronal sections for each experimental group. Sections showing largest tdTomato-positive areas were chosen for each rat. Sizes of tdTomato-positive areas quantified in each section were shown in each image. Scale bar, 300 μm. (B) Distribution of sizes of tdTomato-positive areas in all rats used for spine morphological analysis. The size of the supragranular layer of D1 column roughly estimated as 500 μm × 500 μm (25 × 10^4^ μm^2^) is shown as a broken line on the graph.
(TIF)

## Abbreviations

ADF: actin depolymerizing factor
EDP: experience-dependent plasticity
eGFP: enhanced green fluorescent protein
eYFP: enhanced yellow fluorescent protein
HEK: human embryonic kidney
LTD: long-term depression
MAP2: microtubule-associated protein 2
miRNA: microRNA
WT: wild type
