# Supplementary figures and images for: Cofilin1 Controls Transcolumnar Plasticity in Dendritic Spines in Adult Barrel Cortex
Source: PLoS Biol. 2015 Feb 27;13(2):e1002070. doi: 10.1371/journal.pbio.1002070 (PMC4344332; doi:10.1371/journal.pbio.1002070)

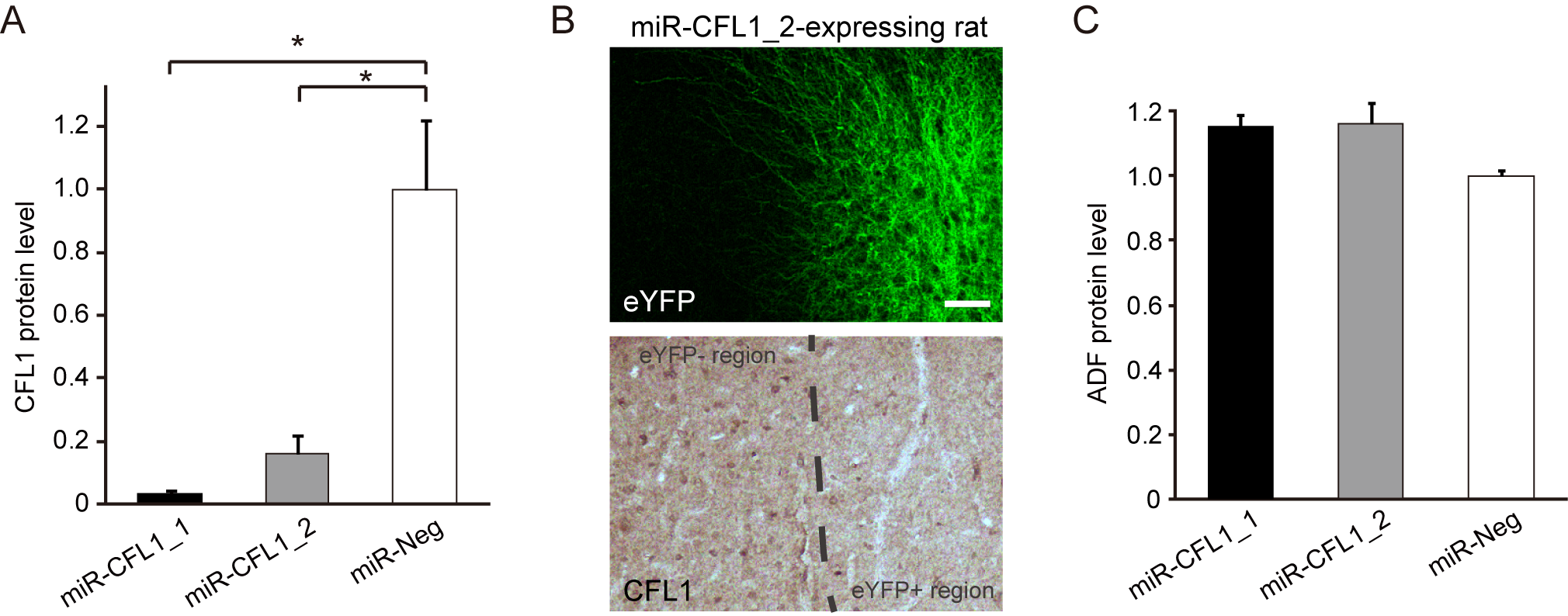

Supplement: S1 Fig — (A) CFL1 protein KD efficiency of miR-CFL1_1 and miR-CFL1_2 in PC-12 cells. CFL1 protein levels were normalized to those of the miR-Neg group. n = 3 for all groups. miR-CFL1_1, p = 0.0036; miR-CFL1_2, p = 0.0073 versus miR-Neg, Dunnett’s multiple comparison test. (B) Two neighboring coronal sections from a miR-CFL1_2 virus-injected rat are shown, one depicting eYFP fluorescence (top) and the other depicting CFL1 immunoreactivity (bottom). (C) Effects of miR-CFL1 on ADF protein expression in PC-12 cells. n = 3 for all groups. miR-CFL1_1, p = 0.094; miR-CFL1_2, p = 0.078: versus miR-Neg. (TIF) [file pbio.1002070.s002.tif]

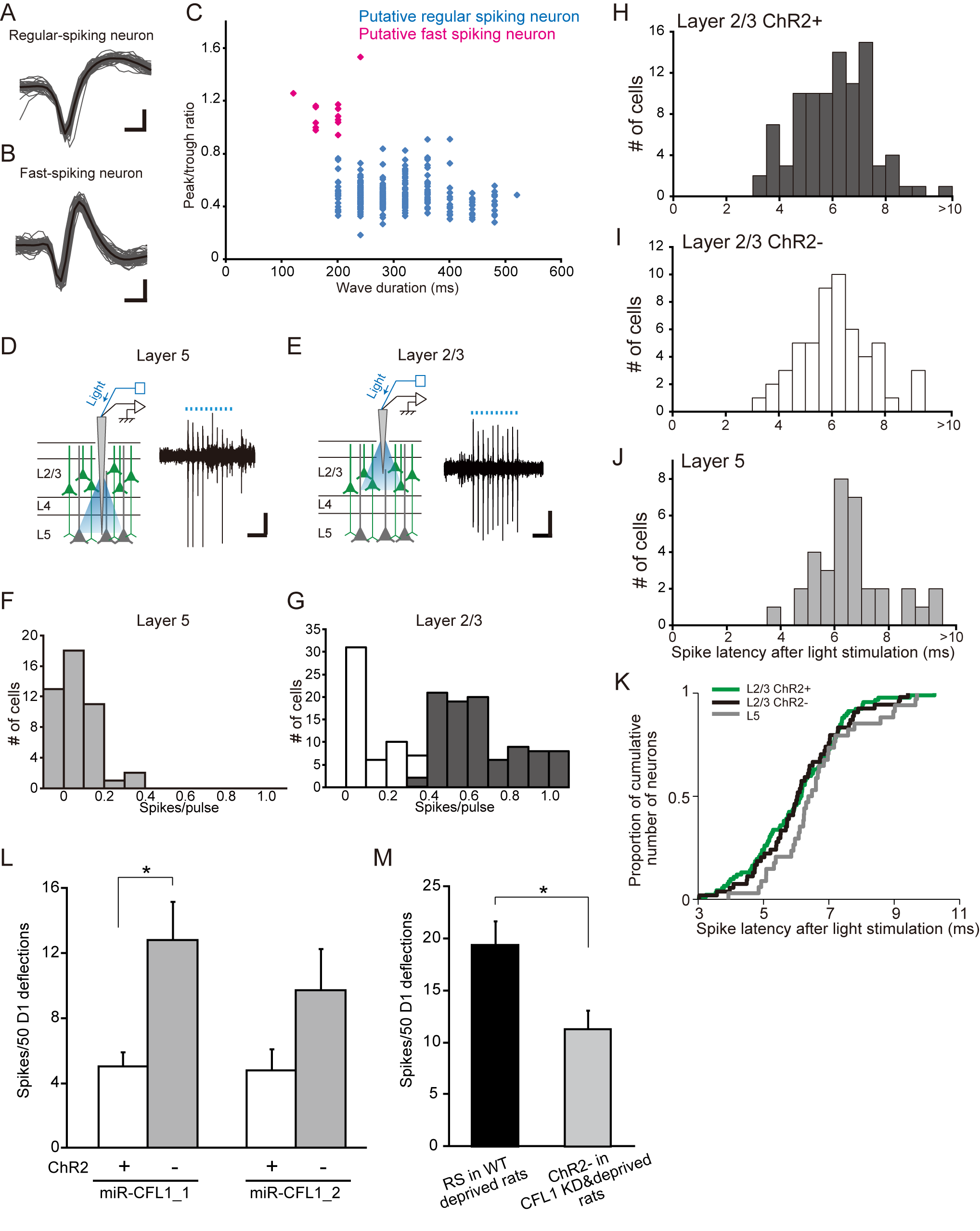

Supplement: S2 Fig — (A, B) Representative waveforms of a regular-spiking neuron (A) and a fast-spiking neuron (B). Gray lines indicate single spike waveforms, and black lines show averaged waveforms (n = 30). Horizontal scale bar, 200 μs; vertical scale bar, 150 μV. (C) Population data. The wave duration (difference between peak and trough time) and peak/trough ratio of the waveform for each neuron are plotted (n = 332). The magenta symbols correspond to neurons that were classified as fast-spiking neurons. (D, E) Representative response patterns of L5 (D) and L2/3 neurons (E) to 20 Hz repetitive light stimulation. Scale bars: horizontal, 200 ms; vertical, 200 μV. (F, G) Distribution of spike probability in light-responsive neurons of L5 (F) and L2/3 neurons (G). The dark gray bars in G correspond to neurons that were classified as putative ChR2+ neurons, and the white bars correspond to neurons that were classified as putative ChR2− neurons. The distribution shown in F was the result of unbiased sampling. By contrast, it is important to note that the distribution shown in G was the result of biased sampling; we searched neurons that showed high response reliability to the repetitive light stimulation online. (H−J) Latency distribution of L2/3 putative ChR2+ (H), L2/3 putative ChR2− (I), and L5 light-responsive neurons (J) (n = 92 (L2/3 ChR2+), 54 (L2/3 ChR2–), and 34 (L5 light-responsive). (K) Cumulative frequency histogram of latency for neuronal population. None of the combination showed significant difference (L2/3 ChR2+ versus L2/3 ChR2−, p = 0.90; L2/3 ChR2+ versus L5 light-responsive, p = 0.16; L2/3 ChR2− versus L5 light-responsive, p = 0.21; Kolmogorov-Smirnov test). (L) Average number of spikes measured in putative ChR2+ and ChR2− L2/3 neurons in response to D1 whisker stimulation for each rat group is shown. n = 23, 28, 18, and 25 units for miR-CFL1_1 ChR2+, miR-CFL1_1 ChR2−, miR-CFL1_2 ChR2+, and miR-CFL1_2 ChR2−, respectively. F 1, 90 = 9.01, p = 0.0035, main effect of fact [file pbio.1002070.s003.tif]

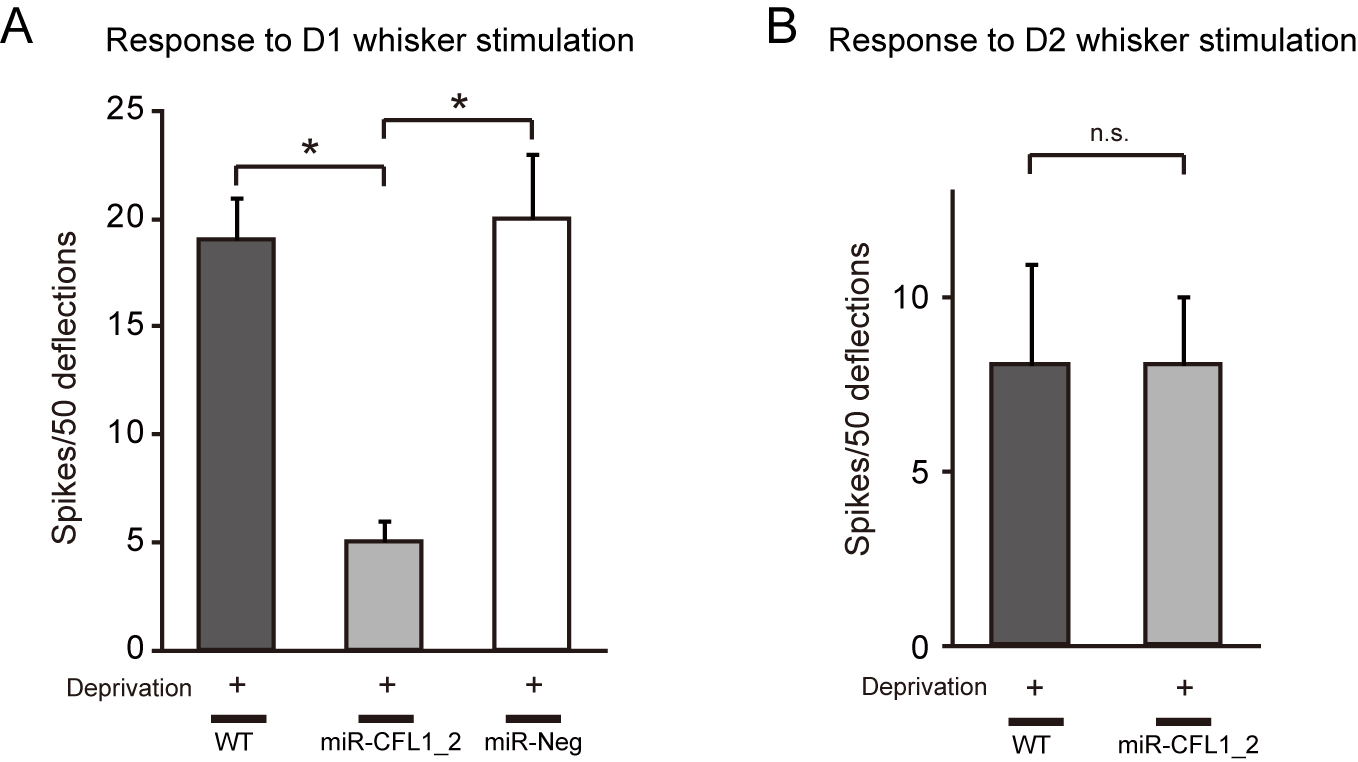

Supplement: S3 Fig — (A) Average number of spikes measured in D2 neurons in response to D1 whisker stimulation for each rat group is shown. n = 33, 18, and 16 units for WT deprived, miR-CFL1_2 deprived, and miR-Neg deprived, respectively. WT deprived versus miR-CFL1_2 deprived, p = 4.1 × 10^-6^, Tukey-Kramer’s multiple comparison test. (B) Average number of spikes measured in D2 neurons in response to D2 whisker stimulation for each rat group. n = 33 and 18 units for WT deprived and miR-CFL1_2 deprived groups, respectively. p = 0.89, Student’s t-test. (TIF) [file pbio.1002070.s004.tif]

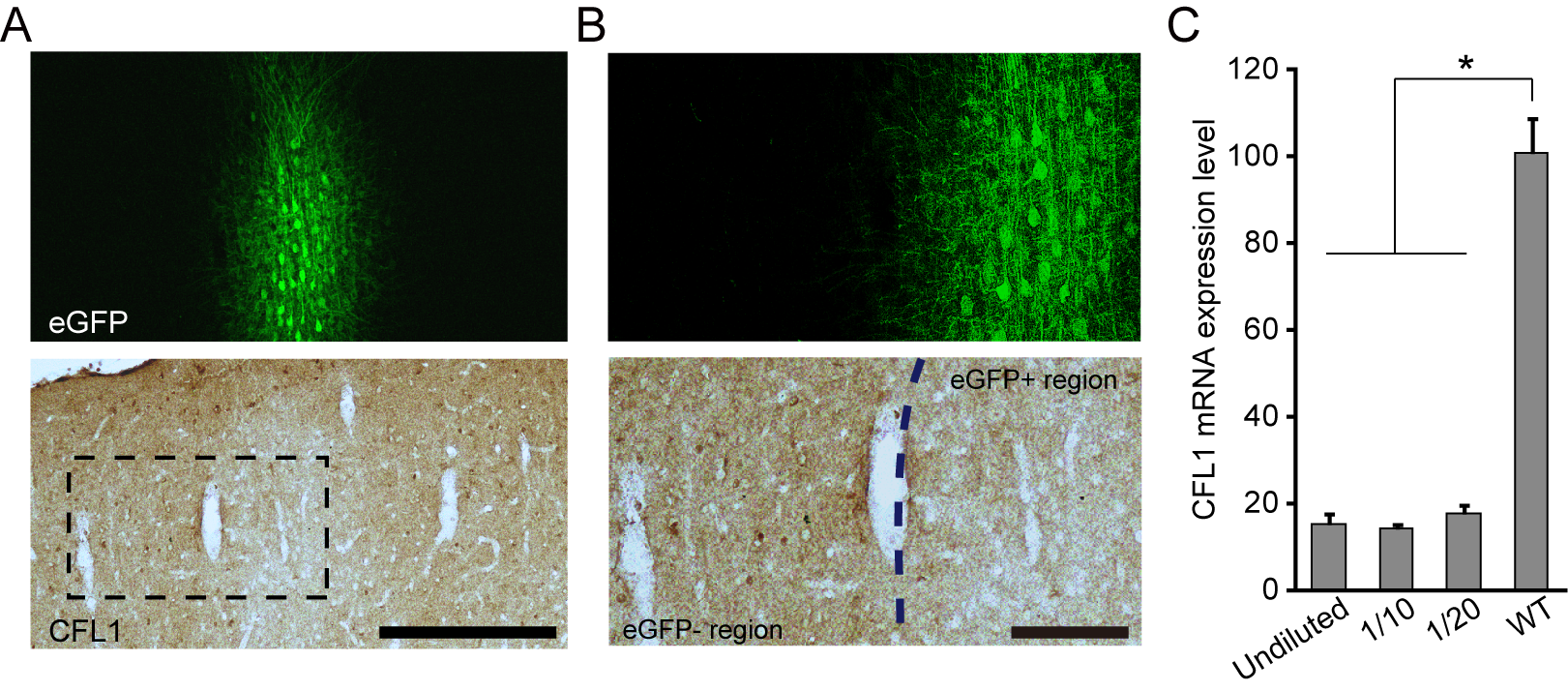

Supplement: S4 Fig — (A) Two neighboring coronal sections from a Lenti-CaMKIIα-eGFP-miR-CFL1_1-injected rat (titer, 6.3 × 10^8^ gc·ml−1) are shown, one depicting eGFP fluorescence (top) and the other depicting CFL1 immunoreactivity (bottom). Scale bar, 300 μm. (B) Magnified view of the rectangular region indicated in (A). Scale bar, 100 μm. (C) Lentiviral titer dependence of KD efficiencies measured in vitro. Rat CFL1-overexpressed HEK293T cells (8.0 × 10^4^ cells) were infected with the miR-CFL1_1 expressing vector (Lenti-CMV-ChR2-eYFP-miR-CFL1_1) in different titer conditions (1.6 × 10^7^, 1.6 × 10^6^, and 8.0 × 10^5^ gc for undiluted, 1/10, and 1/20 groups, respectively). ‘“WT” indicates uninfected cells. n = 3 for all groups. Undiluted, p = 4.1 × 10^-6^; 1/10, p = 3.8 × 10^-6^; 1/20, p = 5.2 × 10^-6^ versus WT, Tukey-Kramer’s multiple comparison test. (TIF) [file pbio.1002070.s005.tif]

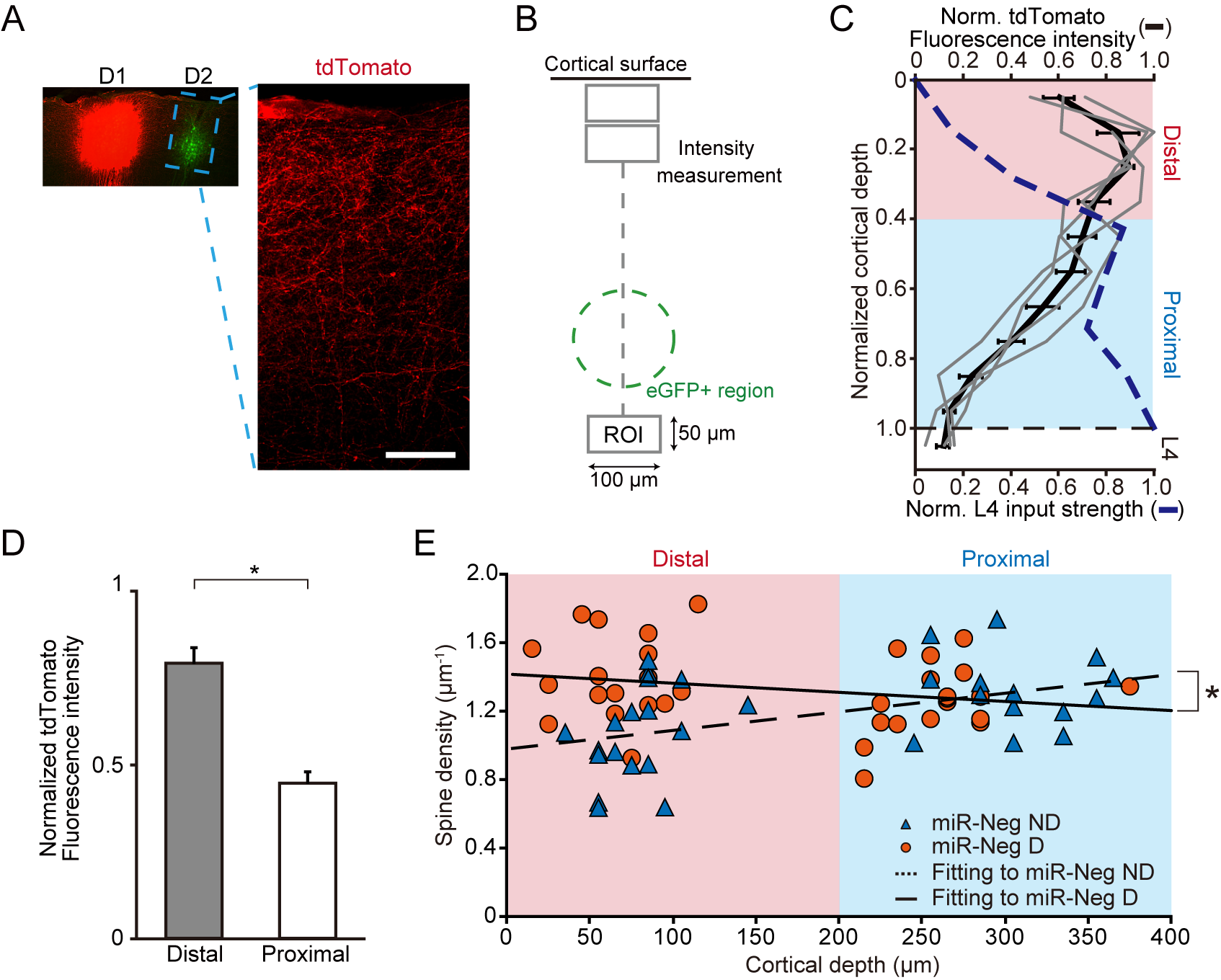

Supplement: S5 Fig — (A) Magnified view of an eGFP-expressing region in the parasagittal section shown in Fig. 6E. Scale bar, 100 μm. (B) The tdTomato fluorescence intensity derived from D1 axons was measured in the rectangular region of interest in the D2 column. The rectangle was vertically scanned within the D2 column. (C) An averaged vertical profile of normalized tdTomato fluorescence intensity (black line). Gray lines indicate the data for each rat (n = 4 rats). See Experimental procedures for details regarding the normalization of fluorescence intensity and cortical depth. A vertical profile of L4 input strength measured in Petreanu and colleagues was also plotted on the same graph [44] (blue dashed line). Based on these observations, the supragranular layer was separated into the distal portion (0–200 μm from the cortical surface) and the proximal portion (200–500 μm), in which horizontal transcolumnar inputs or ascending intracolumnar inputs, respectively, are thought to predominate. (D) The tdTomato fluorescence intensity was averaged in either the distal or proximal portion of the supragranular layer. *p = 0.022, paired t-test. (E) Dendritic spine densities measured around (<15 μm) spines receiving D1 inputs in the miR-Neg non-deprived (ND) and deprived (D) groups were plotted against the cortical depth of the midpoints of the measured dendritic segments (bin width, 50 μm). Each triangle and circle represents a dendritic branch segment for the miR-Neg ND and D groups, respectively. The dashed and solid lines correspond to lines fitted to the distribution of miR-Neg ND and D groups, respectively, by linear regression. F 1,63 = 9.33, *p = 0.0033, F-test (the null hypothesis stated that the slopes of the lines fitted by linear regression were equivalent). (TIF) [file pbio.1002070.s006.tif]

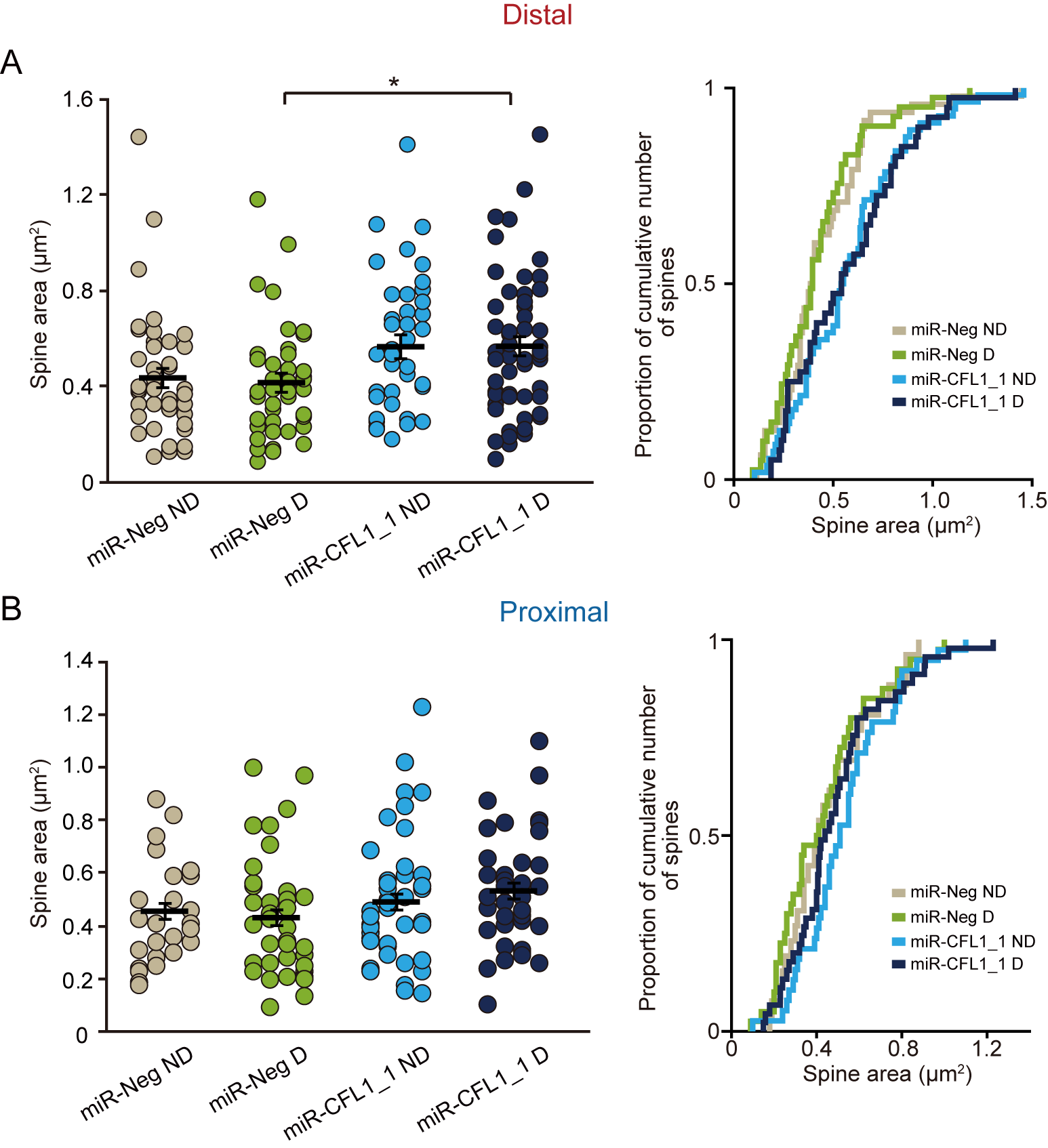

Supplement: S6 Fig — (A) Plots of dendritic spine areas measured in the distal portion of L2/3 for each rat group (left) and cumulative frequency histogram (right). n = 48, 41, 40, and 56 spines for miR-Neg ND, miR-Neg D, miR-CFL1_1 ND, and miR-CFL1_1 D, respectively. F 1, 181 = 10.7, p = 0.0013, main effect of factor 1; factor 1, miR type; factor 2, deprivation type; two-way ANOVA. miR-Neg ND versus miR-CFL1 ND, p = 0.10; miR-Neg ND versus miR-CFL1 D, p = 0.060; miR-Neg D versus miR-CFL1 ND, p = 0.060; miR-Neg D versus miR-CFL1 D, p = 0.033: Tukey-Kramer’s multiple comparison test. miR-Neg ND versus miR-CFL1 ND, p = 0.10; miR-Neg ND versus miR-CFL1 D, p = 0.11; miR-Neg D versus miR-CFL1 ND, p = 0.12; miR-Neg D versus miR-CFL1 D, p = 0.038: Kolmogorov-Smirnov test with Bonferroni’s correction. (B) Same as (A) but of dendritic spine areas measured in the proximal portion. n = 26, 40, 45, and 38 spines for miR-Neg ND, miR-Neg D, miR-CFL1_1 ND, and miR-CFL1_1 D groups, respectively. (TIF) [file pbio.1002070.s007.tif]

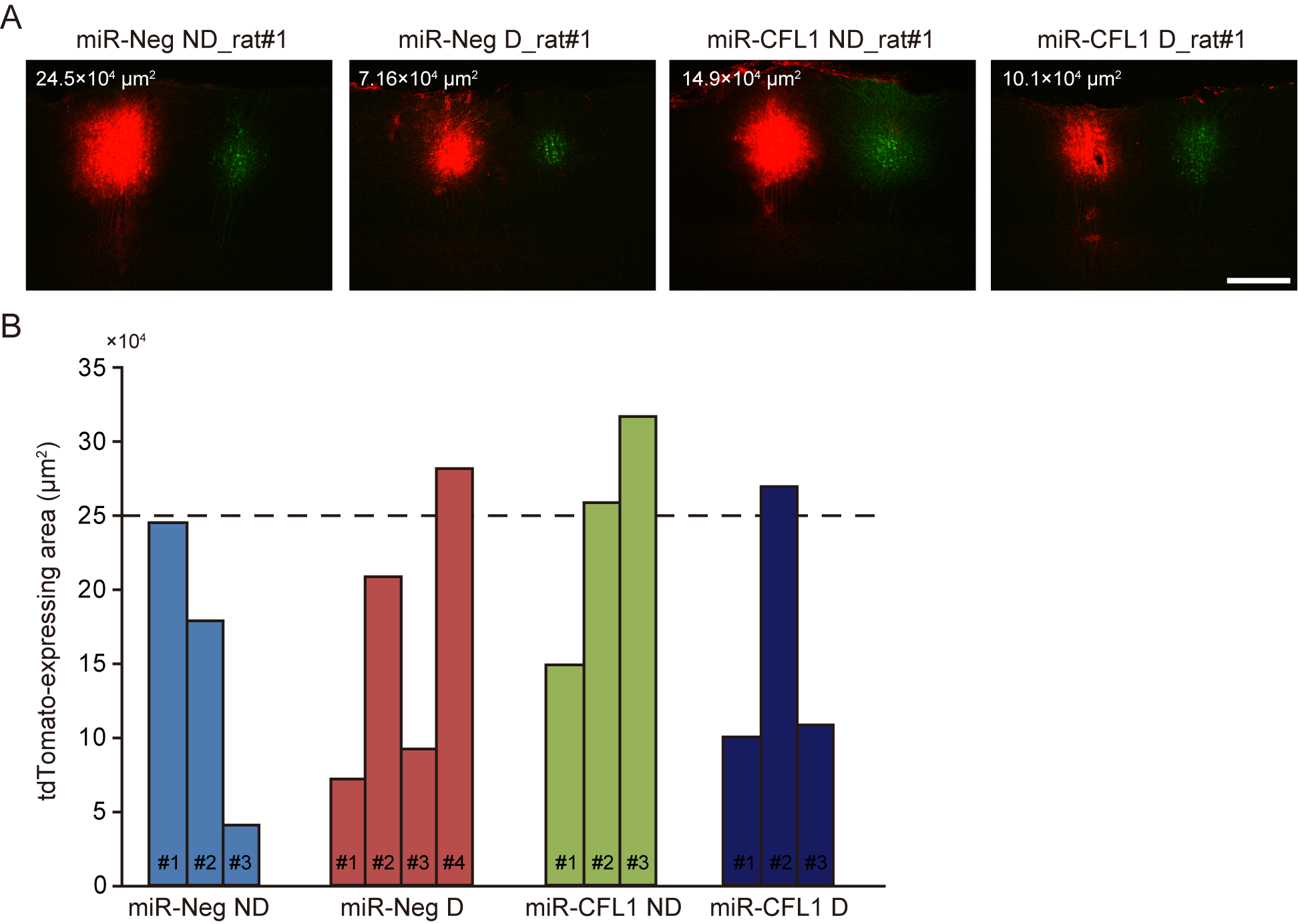

Supplement: S7 Fig — (A) Example coronal sections for each experimental group. Sections showing largest tdTomato-positive areas were chosen for each rat. Sizes of tdTomato-positive areas quantified in each section were shown in each image. Scale bar, 300 μm. (B) Distribution of sizes of tdTomato-positive areas in all rats used for spine morphological analysis. The size of the supragranular layer of D1 column roughly estimated as 500 μm × 500 μm (25 × 104 μm2) is shown as a broken line on the graph. (TIF) [file pbio.1002070.s008.tif]
